# Reduced risk of clinically important deteriorations by ICS in COPD is eosinophil dependent: a pooled *post-hoc* analysis

**DOI:** 10.1186/s12931-020-1280-y

**Published:** 2020-01-10

**Authors:** Mona Bafadhel, Dave Singh, Christine Jenkins, Stefan Peterson, Thomas Bengtsson, Peter Wessman, Malin Fagerås

**Affiliations:** 1 Respiratory Medicine Unit, Nuffield Department of Medicine, University of Oxford, Old Road Campus, Oxford, OX3 7LF UK; 2 Medicines Evaluation Unit, Manchester University NHS Foundation Trust, University of Manchester, Manchester, UK; 3 The George Institute for Global Health, University of New South Wales, Sydney, Australia; 4 StatMind, Lund, Sweden; 5 AstraZeneca, Gothenburg, Sweden

**Keywords:** COPD, Clinically important deterioration (CID), Exacerbations, Budesonide/formoterol, Eosinophils

## Abstract

**Background:**

Clinically Important Deterioration (CID) is a novel composite measure to assess treatment effect in chronic obstructive pulmonary disease (COPD). We examined the performance and utility of CID in assessing the effect of inhaled corticosteroids (ICS) in COPD.

**Methods:**

This *post-hoc* analysis of four budesonide/formoterol (BUD/FORM) studies comprised 3576 symptomatic moderate-to-very-severe COPD patients with a history of exacerbation. Analysis of time to first CID event (exacerbation, deterioration in forced expiratory volume in 1 second [FEV_1_] or worsening St George’s Respiratory Questionnaire [SGRQ] score) was completed using Cox proportional hazards models.

**Results:**

The proportion of patients with ≥1 CID in the four studies ranged between 63 and 77% and 69–84% with BUD/FORM and FORM, respectively, with an average 25% reduced risk of CID with BUD/FORM. All components contributed to the CID event rate. Experiencing a CID during the first 3 months was associated with poorer outcomes (lung function, quality of life, symptoms and reliever use) and increased risk of later CID events. The effect of BUD/FORM versus FORM in reducing CID risk was positively associated with the blood eosinophil count.

**Conclusions:**

Our findings suggest that BUD/FORM offers protective effects for CID events compared with FORM alone, with the magnitude of the effect dependent on patients’ eosinophil levels. CID may be an important tool for evaluation of treatment effect in a complex, multifaceted, and progressive disease like COPD, and a valuable tool to allow for shorter and smaller future outcome predictive trials in early drug development.

## Background

Clinical trials in chronic obstructive pulmonary disease (COPD) usually evaluate treatment effect by assessing improvements in individual outcomes such as lung function and quality of life (QoL). Due to the progressive nature of COPD, the mean improvements in clinical outcome are often small and require large and/or lengthy trials. An alternative is to study the effect of treatment using a composite endpoint of disease deterioration. Clinically Important Deteriorations (CID) is a composite endpoint consisting of three components of COPD worsening: COPD exacerbations, deteriorations in lung function (as measured by forced expiratory volume in 1 second [FEV_1_]), and worsening QoL (as measured by the St George’s Respiratory Questionnaire [SGRQ]) [1]. CID has been shown to predict long-term outcomes, including mortality [2].

Studies using CID have focused primarily on long-acting bronchodilator effects [1, 3–6], mostly in COPD populations without increased exacerbation risk, often leading to a predominance of CID events triggered by lung function deteriorations, and a relatively small contribution of exacerbations to the composite index. Recently, higher blood eosinophil counts have been shown to be associated with increased exacerbation risk in COPD patients not treated with inhaled corticosteroids (ICS) [7, 8]. The relationship between blood eosinophils and risk of CID is unknown.

In this *post-hoc* analysis of four randomized clinical trials (SUN [9], SHINE [10], US3 [11] and RISE [12]), we assessed the effect of an ICS/long-acting β_2_-agonist (LABA) combination, budesonide/formoterol (BUD/FORM), versus the LABA mono-component alone, on the risk of CID in symptomatic patients with COPD and a history of exacerbations. We evaluated the protective effect of ICS on CID, as well as the predictive properties of peripheral blood eosinophil count and other clinical characteristics. We also assessed the prognostic properties of a CID event on the risk of further events and future deterioration in lung function and quality of life, and how this might impact on future clinical trial design.

## Methods

### Study designs and population

Details of the study designs have been published previously [9–12]. Briefly, SUN [9] and US3 [11] were 52-week, and SHINE [10] and RISE [12] were 26-week, multicentre, randomized, double-blind, double-dummy, parallel-group studies. Details regarding study designs can be found in the supplement (Additional file 1: Table S1). Here, we report the results for analyses comparing the twice daily (bid) BUD/FORM 160/4.5 μg pressurized metered dose inhaler (pMDI) and FORM 4.5 μg dry powder inhaler (DPI) treatment arms, a comparison included in all four studies, in a total of 3576 patients.

Patients were aged ≥40 years with a current clinical diagnosis of COPD and were current or former smokers, with a pack-year history of > 10 years. All patients had confirmed airflow obstruction and a history of ≥1 exacerbation. In SUN, SHINE and US3, all current COPD medications, with the exception of ICS, were discontinued during the run-in period. In RISE, all patients were treated with BUD/FORM 160/4.5 μg bid during run-in. Albuterol (salbutamol) was provided for as-needed use.

### Procedures

Detailed demographic data were collected at baseline. All patients had lung function and health status recorded at scheduled visits (Additional file 1: Table S1). Exacerbations were defined per protocol as worsening of COPD that required treatment with a course of systemic corticosteroids with or without antibiotics, or required hospitalization. A differential full blood count was collected at study entry, except in the RISE trial.

### CID definition

A CID event was defined as the occurrence of *any* of the following individual components: a ≥ 100-mL decrease from baseline in pre-dose FEV_1_, a ≥ 4-unit increase from baseline in SGRQ total score, or the start of a moderate-to-severe COPD exacerbation after the first dose of study medication.

### Statistical analyses

Studies were analysed using the full analysis population. Analysis was performed for each study separately, unless otherwise indicated. Analyses were completed until end-of-study and on censored data at 3 months (Day 90) and 6 months (Day 180).

Each first CID was identified by the first occurrence of any CID component, with the day of onset set as the onset day of that event. Patients with no CID events were censored at the last study day. For analysis of individual components, the first occurrence of the component event was used, independent of whether this coincided with the first CID or not.

Time to first CID was displayed using Kaplan-Meier plots, from which median event times were determined. Time to first CID was analysed using Cox proportional hazards models adjusting for treatment and country. BUD treatment effects were expressed as hazard ratios (HR) between BUD/FORM and FORM alone, with 95% confidence intervals and two-sided *p*-values. To test the assumption of proportional hazards (heterogeneous effect over time) assumed in the Cox model, models adjusting for treatment, country and interaction treatment by logarithm of the day of event were used.

Subgroup analyses of time to first CID were performed based on baseline characteristics, including smoking status, long-acting muscarinic antagonists (LAMA) and ICS use prior to study entry, baseline lung function, exacerbation history, age, gender, and total SGRQ score (Additional file 1: Figure S1). The analyses were performed using Cox proportional hazards models adjusting for treatment, strata and treatment by strata interaction.

To explore the predictive value of an early CID event, patients were divided based on occurrence of a first CID within 3 months (84 days; CID+), or not (CID-). For each stratum and study, the mean change from baseline over the full study period for FEV_1_ and SGRQ was constructed using last value carried forward to impute missing data. Mean changes from baseline were constructed for total daily rescue use and total symptoms (sum of Breathlessness, Cough, and Sputum Scale [BCSS©] scores) based on weekly means of daily observations.

The number of CID events per patient was defined as all observed CID events, in which any CID starting or ending within 7 days would be counted as one event. Analysis of the number of CID events used negative binomial modelling adjusting for treatment and country and using the natural logarithm of the time in study as offset. Estimated event rates were annualized and treatment effect was expressed as a relative rate ratio (RR).

The prognostic and predictive properties of baseline blood eosinophil counts were investigated using a cumulative approach that utilized cut-off levels covering the main part of the eosinophil spectra (0.07–0.35 × 10^9^/L with a 0.01 step). For each cut-off level, patients were divided into a lower and higher stratum (≤cut-off and > cut-off, respectively); the analyses from different cut-offs were combined to determine the relationship between eosinophil levels and CID events. The SUN, SHINE, and US3 studies were pooled for analyses, using Cox proportional hazards models adjusting for treatment and stratified by study. HRs from each analysis were plotted versus the cut-off level, showing the change in effect when extending the eosinophil range upwards (lower stratum, left to right) or downwards (upper stratum, right to left). The HR from the analysis of the full population is indicated as a reference to show the convergence point of the curves. A pooled analysis was performed using a negative binomial model for the number of CID events by baseline eosinophil level, with treatment and study as fixed factors.

## Results

Demographic and baseline characteristics were similar between studies (Table 1 and Additional file 1: Table S2), and were similar between patients who did and did not experience a CID during the studies (Additional file 1: Table S3).

**Table 1:** Demographic and baseline characteristics by study (budesonide/formoterol 160/4.5 μg and formoterol 4.5 μg arms only)

Characteristic	SUN (*n* = 989)	SHINE (*n* = 561)	US3 (*n* = 807)	RISE (*n* = 1219)
Age, years	63.0 (40–88)	63.3 (41–89)	63.1 (40–87)	63.5 (40–87)
Male, n (%)	631 (63.8)	374 (66.7)	490 (60.7)	698 (57.3)
Female, n (%)	358 (36.2)	187 (33.3)	317 (39.3)	521 (42.7)
Race, n (%)				
White	914 (92.4)	523 (93.2)	667 (82.8)	1119 (91.8)
Black	23 (2.3)	20 (3.6)	33 (4.1)	39 (3.2)
Asian	5 (0.5)	2 (0.4)	10 (1.2)	17 (1.4)
Other	47 (4.8)	16 (2.9)	96 (11.9)	44 (3.6)
Former smoker, n (%)	573 (57.9)	319 (56.9)	516 (63.9)	655 (53.7)
Current smoker, n (%)	416 (42.1)	242 (43.1)	291 (36.1)	564 (46.3)
No. exacerbations in previous year	1.8 (1–13)	1.6 (0–8)	1.7 (1–12)	1.4 (1–7)
Post-FEV_1_, L	1.18 (0.35–3.26)	1.20 (0.30–3.29)	1.11 (0.34–2.96)	1.38 (0.34–3.6)
Post-FEV_1_, % predicted	38.9 (13–92)	39.3 (10–103)	37.7 (12–77)	48.7 (16–78)
FEV_1_/FVC ratio	0.49 (0.20–0.85)	0.48 (0.20–0.82)	0.47 (0.16–1.00)	0.49 (0.19–0.75)
Eosinophils, ×10^9^/L, geometric mean (range)	0.18 (0.01–1.47)	0.18 (0.01–1.01)	0.13 (0.01–2.51)	N/A^a^
SGRQ total score	54.9 (7–100)	54.9 (14–100)	57.5 (6–99)	46.7 (0–97)

Data presented as mean (range) unless otherwise stated

^a^Laboratory data was not assessed in RISE; therefore, no baseline data are available for eosinophils

*FEV*_*1*_ Forced expiratory volume in 1 s, *FVC* Forced vital capacity, *N/A* Not applicable, *SGRQ* St George’s Respiratory Questionnaire

For full baseline characteristics, see Additional file 1: Table S2

### CID events

**Fig. 1:**
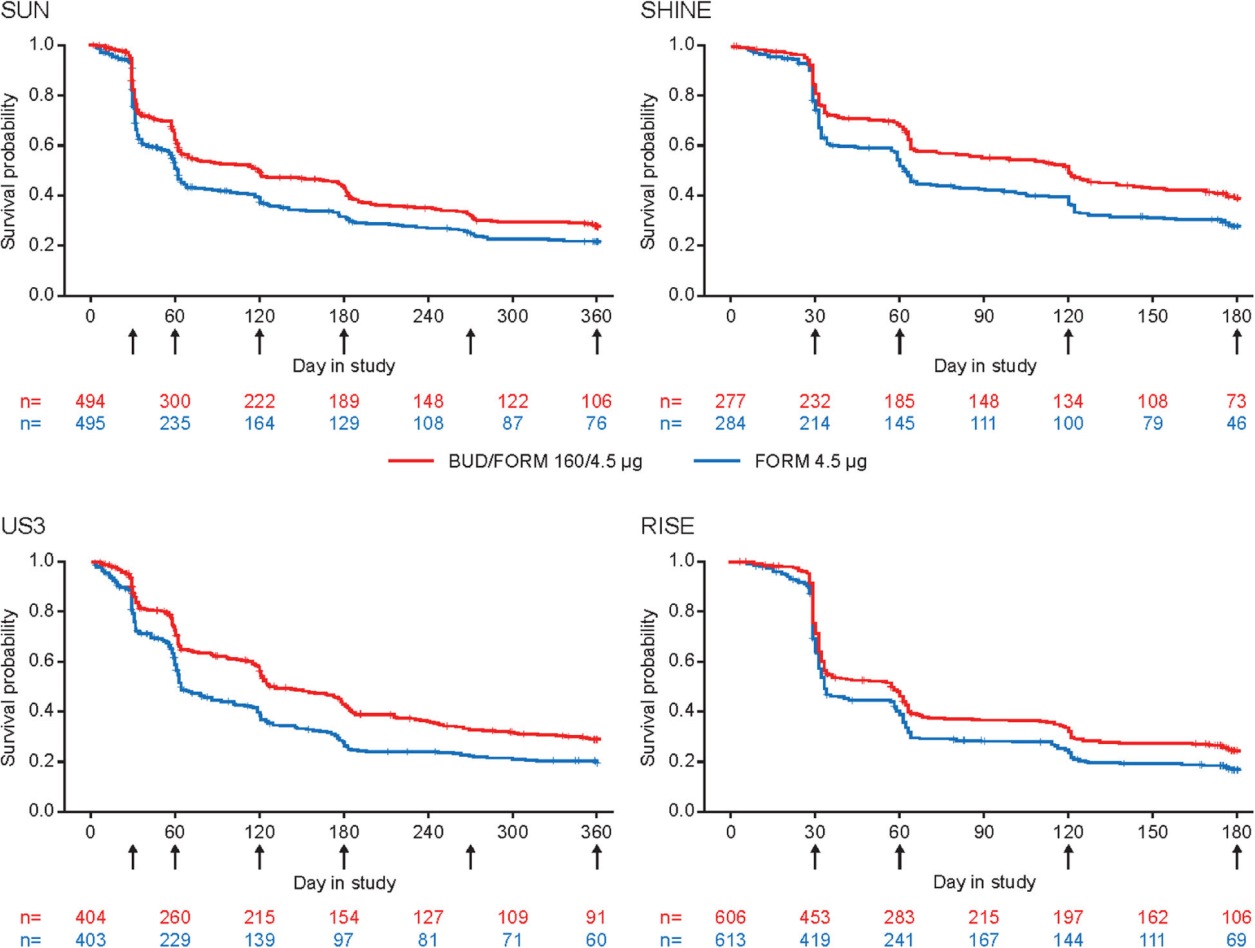
Kaplan–Meier curves for time to first CID, by study. BUD, budesonide; CID, Clinically Important Deterioration; FORM, formoterol. Arrows indicate clinical visits

The proportion of patients experiencing ≥1 CID over the study duration ranged from 63 to 77% and 69–84% in the BUD/FORM and FORM arms, respectively, across the four studies. The majority of CID events were triggered by one criterion only, with ≤15% of the first CID event fulfilling more than one criterion. Events fulfilling the FEV_1_ criterion accounted for 35–50% of the first CID events, SGRQ criterion 26–50%, and exacerbation criterion 17–45% in the studies (Table 2). Due to the definition of CID, many events were clustered around the clinical visits (Fig. 1).

**Table 2:** Summary of events and characteristics by study

	SUN (n = 989)		SHINE (n = 561)		US3 (n = 807)		RISE (n = 1219)	
	BUD/FORM 160/4.5 μg bid (*n* = 494)	FORM 4.5 μg bid (*n* = 495)	BUD/FORM 160/4.5 μg bid (*n* = 277)	FORM 4.5 μg bid (*n* = 284)	BUD/FORM 160/4.5 μg bid (*n* = 404)	FORM 4.5 μg bid (*n* = 403)	BUD/FORM 160/4.5 μg bid (*n* = 606)	FORM 4.5 μg bid (*n* = 613)
Any CID	343 (69.4)	360 (72.7)	173 (62.5)	196 (69.0)	280 (69.3)	305 (75.7)	468 (77.2)	517 (84.3)
Subtypes of first CID								
Exacerbation alone	81 (23.6)	96 (26.7)	47 (27.2)	47 (24.0)	114 (40.7)	126 (41.3)	61 (13.0)	74 (14.3)
FEV_1_ event alone	120 (35.0)	112 (31.1)	62 (35.8)	59 (30.1)	90 (32.1)	86 (28.2)	172 (36.8)	174 (33.7)
SGRQ alone	116 (33.8)	112 (31.1)	49 (28.3)	76 (38.8)	61 (21.8)	75 (24.6)	172 (36.8)	171 (33.1)
Exacerbation + FEV_1_	3 (0.9)	5 (1.4)	0 (0.0)	2 (1.0)	6 (2.1)	8 (2.6)	5 (1.1)	7 (1.4)
Exacerbation + SGRQ	3 (0.9)	4 (1.1)	4 (2.3)	2 (1.0)	1 (0.4)	4 (1.3)	6 (1.3)	7 (1.4)
FEV_1_ + SGRQ	20 (5.8)	30 (8.3)	10 (5.8)	10 (5.1)	7 (2.5)	5 (1.6)	51 (10.9)	79 (15.3)
Exacerbation + FEV_1_ + SGRQ	0 (0)	1 (0.3)	1 (0.6)	0 (0)	1 (0.4)	1 (0.3)	1 (0.2)	5 (1.0)
Individual components								
Exacerbations	152 (30.8)	177 (35.8)	69 (24.9)	78 (27.5)	169 (41.8)	182 (45.2)	151 (24.9)	181 (29.5)
FEV_1_ events	196 (39.7)	212 (42.8)	84 (30.3)	100 (35.2)	135 (33.4)	161 (40.0)	289 (47.7)	344 (56.1)
SGRQ events	188 (38.1)	202 (40.8)	81 (29.2)	116 (40.8)	143 (35.4)	164 (40.7)	312 (51.5)	357 (58.2)

Event rates in percentage of total number of patients; CID sub-events in percentage of total CID events

The rows with individual components (exacerbations, FEV_1_ events, SGRQ events) describe events as independent variables, whereas the subtypes of first CID events describe the variable/combination occurring first within a patient. Bid, twice daily; *BUD* Budesonide, *CID* Clinically Important Deterioration, *FEV*_*1*_ Forced expiratory volume in 1 s, *FORM* Formoterol, *SGRQ* St George’s Respiratory Questionnaire

### Treatment effect of BUD/FORM

**Fig. 2:**
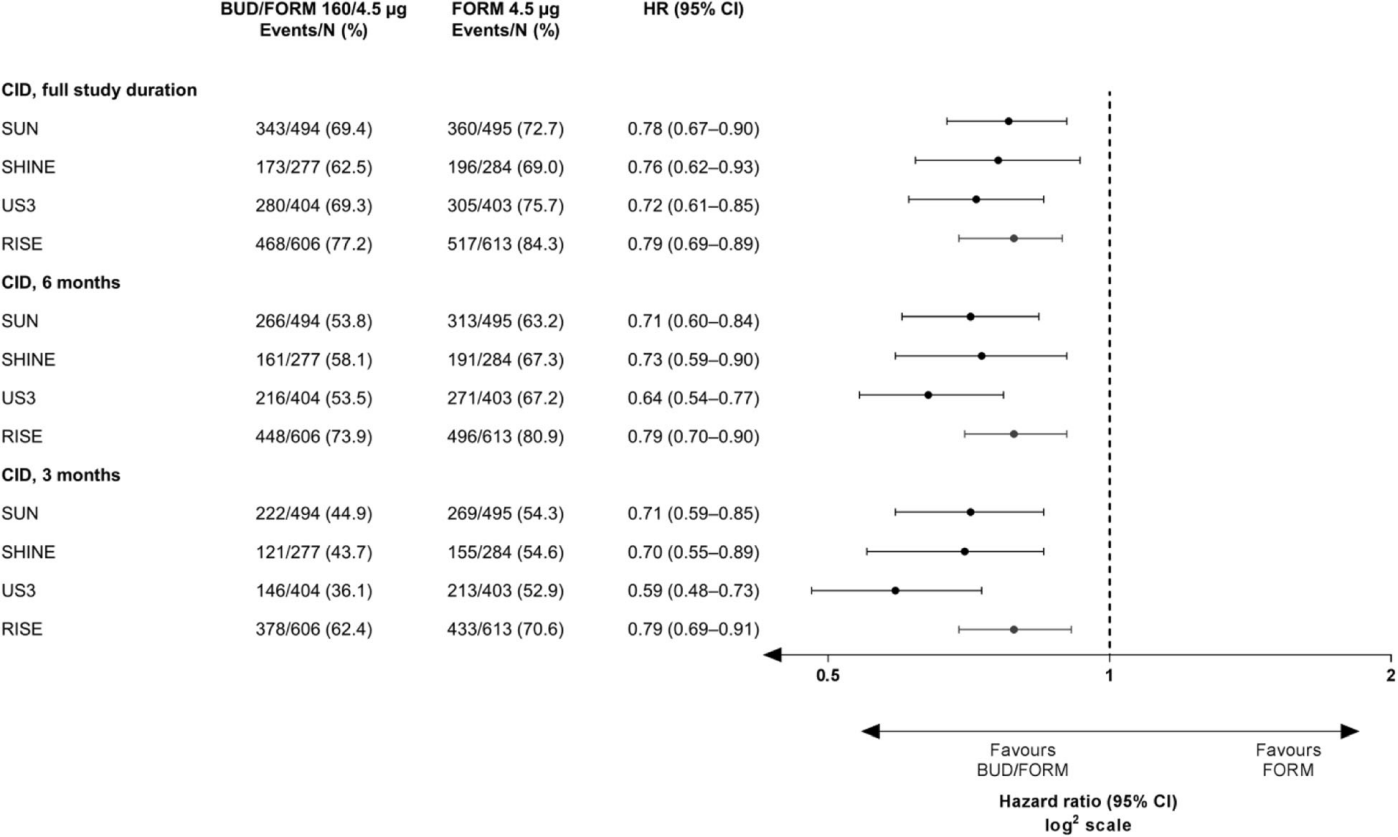
Treatment effect of budesonide added to formoterol on time to first CID by study. Treatment effect of budesonide (BUD) added to formoterol (FORM) on time to first CID by study (full study duration and censored at 6 months and 3 months); an early CID, within the first 3–6 months of study commencement, has been suggested to be predictive of future outcomes such as mortality, lung function decline rate and exacerbations. Therefore, data primarily based on full study length was analysed, but analyses were also conducted on data censored at 6 months (Day 180) and 3 months (Day 90). BUD, budesonide; CID, Clinically Important Deterioration; CI, confidence interval; FORM, formoterol; HR, hazard ratio

Treatment with BUD/FORM significantly prolonged the time to first CID and reduced the risk of CID by 21–28% versus FORM alone in all four studies (*p* < 0.001, Fig. 2). The majority of patients experienced their first CID event early, with median time to first CID of 119–127 days (BUD/FORM) and 62–64 days (FORM) in SUN, SHINE and US3, and 57 days (BUD/FORM) and 33 days (FORM) in RISE (Fig. 1). In all studies, the estimated treatment effect for the individual components were all in favour of BUD/FORM, with estimated risk reductions ranging from 15 to 23% for FEV_1_, 14–39% for SGRQ deteriorations and 20–24% for exacerbations (Additional file 1: Figure S2). Due to fewer events, and thereby lower power, risk reductions did not always reach statistical significance for the individual components.

Tests of the assumptions of proportional hazards for CID indicated significant deviation in the 12-month SUN and US3 studies (*p* = 0.006 and *p* = 0.001, respectively), but not in the 6-month SHINE and RISE studies (*p* = 0.096 and *p* = 0.570, respectively) (Additional file 1: Table S4). This would indicate a non-constant treatment effect over the study period; however, the Kaplan-Meier curves do not cross in either of the studies.

Data analysis with values censored at 3 and 6 months was performed. BUD/FORM reduced the risk of CID between 21 and 41% over 3 months and between 21 and 36% over 6 months versus FORM (Fig. 2). Assessment over the shorter study durations demonstrated improved proportionality of the hazards for both CID and the individual components (Additional file 1: Table S4).

### Early CID versus later CID outcomes

**Fig. 3:**
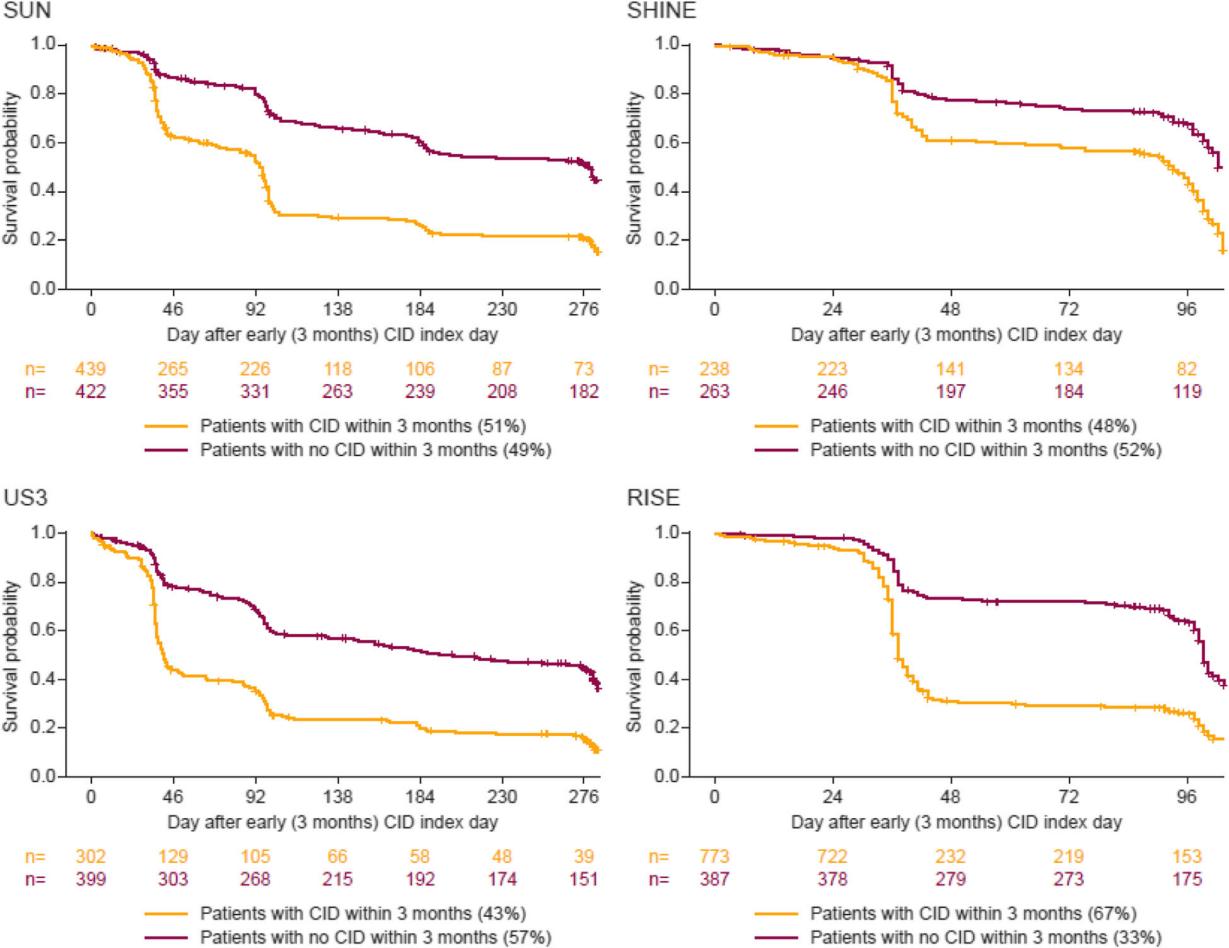
Time to CID event in patients with or without CID in the first 3 months. CID, Clinically Important Deterioration

In the pooled treatment arms, 43–67% of patients experienced ≥1 CID during the first 3 months, termed early CID (CID+). CID+ patients were more likely to experience a further CID after the first 3 months compared with patients without an early CID event (CID-) (Fig. 3).

**Fig. 4:**
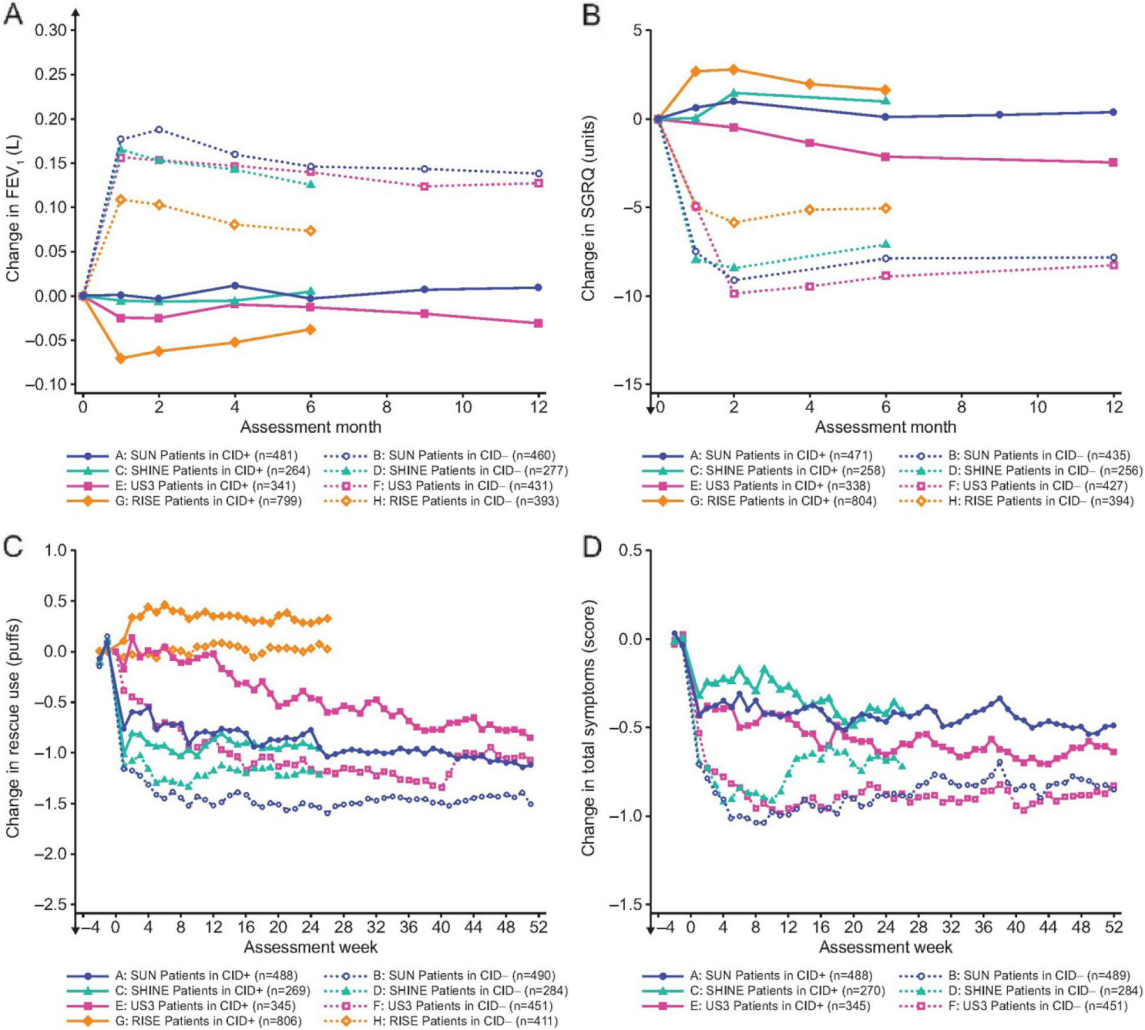
Mean change from baseline in patients with early (CID+) or late (CID-) CID events. Mean change from baseline over the full study duration in A) FEV_1_, B) SGRQ, C) reliever use and D) symptoms in patients with early (CID+) or late (CID-) CID events. Total symptoms (sum of Breathlessness, Cough, and Sputum Scale [BCSS©] scores) were based on weekly means of daily observations. BUD, budesonide; CID, Clinically Important Deterioration; FEV_1_, forced expiratory volume in 1 second; FORM, formoterol; SGRQ, St George’s Respiratory Questionnaire

An early and sustained improvement from baseline in FEV_1_ and SGRQ was observed in CID- patients, with a mean change in FEV_1_ ranging 91–159 mL and a mean change in SGRQ score ranging from − 9.1 to − 5.2 units between studies (Fig. 4a and b). In CID+ patients, FEV_1_ and SGRQ remained similar to baseline values or worsened over the study, with a change from baseline ranging from − 56 to 4 mL in FEV_1_ and from − 1.6 to 2.3 units in SGRQ score (Fig. 4a and b). Compared with baseline, the mean weekly reliever use was reduced in both CID+ and CID- patients, except in RISE (Fig. 4c). Mean weekly total symptom score improved in both CID- and CID+ patients, but to a larger extent in CID- (Fig. 4d).

### Effect of blood eosinophils

**Fig. 5:**
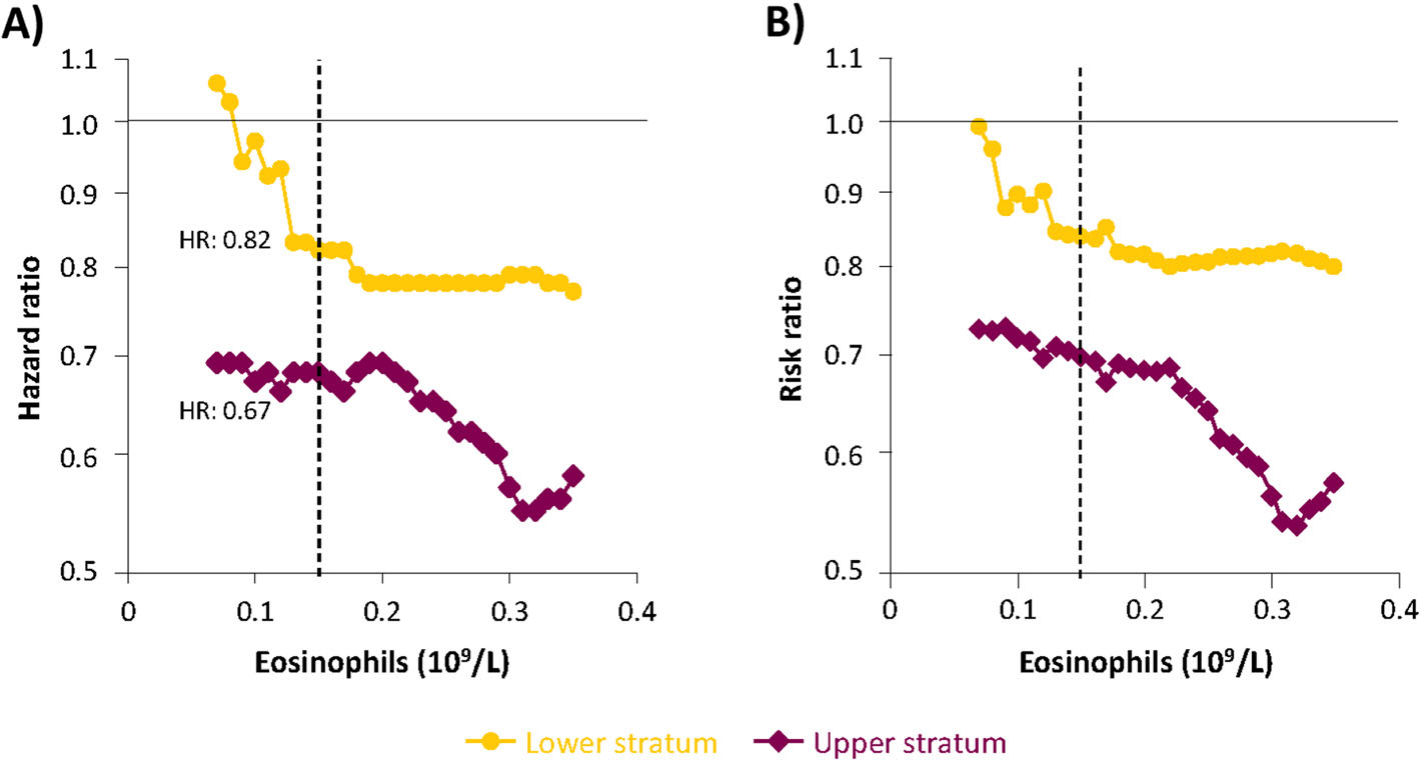
Effect size by eosinophil cut-off in pooled analysis of SUN, SHINE and US3. Effect size by eosinophil cut-off in pooled analysis of SUN, SHINE and US3 for A) time to first CID event (hazard ratio [HR]) and B) number of events (risk ratio [RR]). Note: The solid line represents HR or RR of 1.0, while the dotted line represents treatment effect for the whole population. At each individual eosinophil count plotted, HR is calculated for the lower stratum in yellow (indicating the mean HR for all patients with an eosinophil count at or below cut-off) and the upper stratum in purple (indicating the mean HR for all patients above the cut-off). Example HR presented for cut-off at 0.15 x10^9^/L (vertical dotted line). The eosinophil range used here is narrower than the actual eosinophil range. There were a relatively small number of patients with higher values at a cut-off of 0.36 x10^9^/L, thus relevant analyses cannot be obtained in the upper strata if it were extended beyond this cut off

Baseline blood eosinophil count was available from 2286 patients in SUN, SHINE and US3. When analysing subgroups of lower and upper eosinophil strata, defined based on baseline eosinophil levels below (lower eosinophil stratum) and above (upper eosinophil stratum) a range of cut-offs, the treatment effects (HR) on CID events between BUD/FORM and FORM were consistently greater for patients in the upper stratum compared with patients in the lower stratum (Fig. 5). The 0.10 × 10^9^/L eosinophil cut-off corresponded to a mean HR for BUD/FORM versus FORM of 0.67 (95% CI: 0.60–0.75) for the upper stratum population (above 0.10 × 10^9^/L eosinophils, 75% of patients) while no reduced CID risk of BUD/FORM (HR 0.97; 95% CI: 0.80–1.18) was apparent in the lower stratum (at or below 0.10 × 10^9^/L eosinophils, 25% of patients). The 0.30 × 10^9^/L eosinophil cut-off corresponded to a mean HR for BUD/FORM versus FORM of 0.57 (95% CI: 0.46–0.71) in the upper stratum (20% of patients) compared to a HR of 0.79 (95% CI: 0.71–0.88) in the lower stratum. A similar relationship between baseline eosinophil counts (upper and lower strata) and treatment effect was observed for the individual components of CID (Additional file 1: Figure S3). A selected range of eosinophil cut-offs and corresponding HRs are presented in Table 3. Eosinophil stratum censored data at 3 and 6 months are presented in the supplement (Additional file 1: Figure S4; Additional file 1: Tables S5 and S6).

**Table 3:** Analysis of time to first CID by selected eosinophil cut-offs (pooled analysis; full study duration)

Cut-off, ×10^9^/L (% ≤)	Lower stratum			Upper stratum		
	BUD/FORM 160/4.5 μg bid	FORM 4.5 μg bid	HR (95% CI)	BUD/FORM 160/4.5 μg bid	FORM 4.5 μg bid	HR (95% CI)
0.10 (25.2)	201/285 (70.5)	199/291 (68.4)	0.97 (0.80–1.18)	569/857 (66.4)	633/853 (74.2)	0.67 (0.60–0.75)
0.15 (44.8)	350/503 (69.6)	373/520 (71.7)	0.82 (0.71–0.95)	420/639 (65.7)	459/624 (73.6)	0.68 (0.59–0.77)
0.20 (60.9)	466/675 (69.0)	519/718 (72.3)	0.78 (0.69–0.88)	304/467 (65.1)	313/426 (73.5)	0.69 (0.59–0.80)
0.25 (73.1)	560/818 (68.5)	613/852 (71.9)	0.78 (0.69–0.87)	210/324 (64.8)	219/292 (75.0)	0.64 (0.53–0.78)
0.30 (79.9)	616/896 (68.8)	670/931 (72.0)	0.79 (0.71–0.88)	154/246 (62.6)	162/213 (76.1)	0.57 (0.46–0.71)
0.35 (85.2)	659/965 (68.3)	708/983 (72.0)	0.77 (0.69–0.86)	111/177 (62.7)	124/161 (77.0)	0.58 (0.45–0.75)

Numbers shown: event/total (%)

bid, twice daily; *BUD* Budesonide, *CID* Clinically Important Deterioration, *CI* Confidence interval, *FORM* Formoterol, *HR* Hazard ratio

### Annualized rate of CID

The proportion of patients who experienced > 1 CID over the study period ranged from 33 to 65% (Additional file 1: Figure S5), and the mean number of CID events per patient per year ranged from 2.05–4.27 in the BUD/FORM arm and 2.85–5.04 in the FORM arm in the four studies. The rate ratio for BUD/FORM versus FORM indicated a reduction of 15–28% in the four studies (*p* ≤ 0.001, Additional file 1: Figure S6). A similar size of treatment effect on the individual components was observed (Additional file 1: Figure S6). The relationship between baseline eosinophil levels and the RR, based on analysis of total number of CID, showed a reduced RR at every eosinophil level in the upper stratum compared with the lower stratum (Additional file 1: Figure S7). In patients with blood eosinophil counts < 0.10 × 10^9^/L, low-to-no treatment effect was observed (RR = 0.87; 95% CI: 0.73–1.05).

## Discussion

This analysis demonstrates a protective effect of ICS on CID, with a risk reduction of approximately 25% across the four studies. ICS had a protective effect on all individual CID components, indicating that the benefit offered by BUD extends beyond the effect on exacerbations, and includes prevention of deteriorations in lung function and QoL. This contrasts with the modest effect of ICS when assessing improvements in FEV_1_ and SGRQ previously reported with ICS [13], suggesting that ICS in COPD are primarily preventive and protective, rather than to induce improvements in lung function and QoL only. Patients who experienced a CID within the first 3 months had an increased risk of experiencing additional events later, and had a poorer outcome with regard to lung function decline, worsened QoL, increased reliever use and symptoms over the study period compared with patients who did not experience a CID within the first 3 months. This suggests that CID events are prognostic of longer-term clinical outcomes in patients at increased risk of exacerbations.

The reduced risk of CID by BUD/FORM versus FORM alone was associated with baseline blood eosinophil counts, with a larger effect observed with higher eosinophil counts. These results add to the evidence showing that blood eosinophils are a biomarker which identify patients most likely to benefit from ICS treatment through prevention of exacerbations [7, 8, 14]. Our data also support the use of eosinophils as a predictive biomarker for ICS effects on lung function and QoL, as previously reported in the INCONTROL study, in which higher eosinophil counts were associated with ICS-induced improvements in FEV_1_ and SGRQ scores [7]. Moreover, our data suggest that in patients with low blood eosinophils counts (< 0.1 × 10^9^ cells/L), the treatment benefit of BUD/FORM versus FORM – and thus the effect of BUD – is poor to minimal. This indicates a subpopulation of COPD patients who should be both considered for exclusion from clinical trials investigating the effect of ICS, and in whom there is an unlikely benefit clinically and a potential increase in harm [7, 14]. These findings align with the recommendations in the updated GOLD 2019 report [15]. The data also show that, in patients with an eosinophil count ≥0.1 × 10^9^ cells/L, corresponding to approximately 75% of the study population, BUD reduced the risk of CID by at least 33%, with increasingly beneficial effect with higher eosinophil levels.

Most previous publications on CID in COPD evaluate the effect of bronchodilators [1, 3–6] and, due to the study designs and patient populations, FEV_1_ deterioration is then the most frequent event type reported [9–12]. If FEV_1_ is assessed at all visits, but not SGRQ, this can give an imbalance in the number of individual CID components. When designing new studies with CID as an outcome measure, our findings suggest that study visits should be spread out evenly during the study period, and FEV_1_ and SGRQ should always be assessed simultaneously. In our study we found that the individual CID components contributed substantially to the total CID rate, with the effects of BUD/FORM versus FORM on CID generally reflected by the individual components. A *post-hoc* analysis of the FLAME study [16], in which the effect of indacaterol/glycopyrronium versus salmeterol/fluticasone on CID was evaluated in patients with an exacerbation history, showed a greater effect of the dual bronchodilator on CID prevention. The largest effect was seen on FEV_1_ deteriorations, consistent with other bronchodilator studies [1, 3–6]. Ideally, all components should contribute to the treatment effect in the same direction and with a similar weight. Longer duration studies often record FEV_1_ and SGRQ more frequently near the beginning; this can result in a higher number of FEV_1_ and SGRQ events recorded early on and more exacerbations thereby being censored by such events regarding first CID.

An early CID within the first 3–6 months of study commencement has been suggested to be predictive of future outcomes such as mortality, lung function deterioration and exacerbations [15]. Here, we confirm the potential prognostic value of an early CID by showing that a CID during the first 3 months increases the likelihood of experiencing additional disease deteriorations in the following 3–9 months. In previous reports evaluating treatment effect on CID, only the first event occurrence has been captured with time-to-first analyses [1, 3–6]. Our findings show that a significant number of patients experience a CID (50% already at 4 months), suggesting that studies with shorter duration could focus on time-to-first CID analysis, and that longer studies could concentrate on the total number of CID events. Altogether, this suggests that CID could be an important tool during early clinical drug development, allowing shorter and/or smaller trials compared with traditional exacerbation or lung function trials, while also being predictive of future outcomes. CID offers increased development efficiency, while exposing fewer patients to novel compounds with as yet unknown safety profiles.

In conclusion, the addition of BUD to FORM reduced the risk and number of CID in moderate-to-severe COPD patients; an effect on exacerbations was seen as well as an effect on deteriorations in lung function and QoL, and benefits were apparent for patients with blood eosinophils above 0.1 × 10^9^/L. This indicates that treatment with BUD offers important preventive and protective effects on several important aspects of COPD. Our upper and lower stratum analytical approaches highlight the importance of blood eosinophils to identify patients for ICS treatment with relevance to studies and study design exploring the effect of ICS in COPD. We suggest that CID is a valuable tool for evaluation of treatment effects that address several aspects of a complex, multifaceted and progressive disease like COPD, and could allow for shorter and smaller trials predictive of future outcome for early drug development.

## Supplementary information

**Additional file 1 **: **Table S1**. Visit and assessment schedule by study. **Table S2**. Demographic and baseline characteristics by study (budesonide/formoterol 160/4·5 μg and formoterol 4·5 μg arms only). **Table S3**. Demographic and baseline characteristics by occurrence of CID event by study (budesonide/formoterol 160/4·5 μg and formoterol 4·5 μg arms only). **Table S4**. Outcome of tests on proportional hazards between treatments. **Table S5**. Analysis of time to first CID by selected eosinophil cut-offs (pooled analysis; 3-month data). **Table S6**. Analysis of time to first CID by selected eosinophil cut-offs (pooled analysis; 6-month data). **Figure S1**. Summary of CID events by subgroup and by study: a) patient demographics; b) disease history and c) lung function. **Figure S2**. Forest plot indicating treatment effect of budesonide (BUD) added to formoterol (FORM) on individual components of CID by study, a) full study duration, b) 6 months and c) 3 months. **Figure S3**. Hazard ratios (HRs) by eosinophil cut-off in pooled analysis of SUN, SHINE and US3 for a) CID, b) exacerbations, c) FEV_1_ and d) SGRQ. **Figure S4**. Effect size by eosinophil cut-off in pooled analysis of SUN, SHINE and US3 for a) 6 months and b) 3 months. **Figure S5**. Proportion of patients with CID events, by study. **Figure S6**. Forest plot for rate ratio for CID and individual components, by study. **Figure S7**. Risk ratios by eosinophil cut-off in pooled analysis of SUN, SHINE and US3.

## Data Availability

Data underlying the findings described in this manuscript may be obtained in accordance with AstraZeneca’s data sharing policy described at https://astrazenecagrouptrials.pharmacm.com/ST/Submission/Disclosure.

## Abbreviations

BCSS: Breathlessness, Cough and Sputum Scale
BUD/FORM: Budesonide/Formoterol
CID: Clinically Important Deteriorations
COPD: Chronic Obstructive Pulmonary Disease
DPI: Dry Powder Inhaler
FEV_1_: Forced Expiratory Volume in one second
HR: Hazard Ratios
ICS: Inhaled Corticosteroids
LABA: Long-Acting β_2_-Agonist
LAMA: Long-Acting Muscarinic Antagonists
pMDI: Pressurized Metered Dose Inhaler
QoL: Quality of Life
RR: Rate Ratio
SGRQ: St George’s Respiratory Questionnaire
