# Supplementary material for: Reduced risk of clinically important deteriorations by ICS in COPD is eosinophil dependent: a pooled post-hoc analysis
Source: Respir Res. 2020 Jan 10;21:17. doi: 10.1186/s12931-020-1280-y (PMC6954504; doi:10.1186/s12931-020-1280-y)
Supplement: Supplementary file 1 — Additional file 1 : Table S1. Visit and assessment schedule by study. Table S2. Demographic and baseline characteristics by study (budesonide/formoterol 160/4·5 μg and formoterol 4·5 μg arms only). Table S3. Demographic and baseline characteristics by occurrence of CID event by study (budesonide/formoterol 160/4·5 μg and formoterol 4·5 μg arms only). Table S4. Outcome of tests on proportional hazards between treatments. Table S5. Analysis of time to first CID by selected eosinophil cut-offs (pooled analysis; 3-month data). Table S6. Analysis of time to first CID by selected eosinophil cut-offs (pooled analysis; 6-month data). Figure S1. Summary of CID events by subgroup and by study: a) patient demographics; b) disease history and c) lung function. Figure S2. Forest plot indicating treatment effect of budesonide (BUD) added to formoterol (FORM) on individual components of CID by study, a) full study duration, b) 6 months and c) 3 months. Figure S3. Hazard ratios (HRs) by eosinophil cut-off in pooled analysis of SUN, SHINE and US3 for a) CID, b) exacerbations, c) FEV1 and d) SGRQ. Figure S4. Effect size by eosinophil cut-off in pooled analysis of SUN, SHINE and US3 for a) 6 months and b) 3 months. Figure S5. Proportion of patients with CID events, by study. Figure S6. Forest plot for rate ratio for CID and individual components, by study. Figure S7. Risk ratios by eosinophil cut-off in pooled analysis of SUN, SHINE and US3. [file 12931_2020_1280_MOESM1_ESM.docx]

**Supplementary material**

**Evaluation of Clinically Important Deteriorations and peripheral blood eosinophils: impact on clinical outcomes and study design in patients with chronic obstructive pulmonary disease**

Mona Bafadhel^1^, Dave Singh^2^, Christine Jenkins^3^, Stefan Peterson^4^, Thomas Bengtsson^4^, Peter Wessman^5^,
Malin Fagerås^5^

^1^Respiratory Medicine Unit, Nuffield Department of Medicine, University of Oxford, Oxford, UK;
^2^Medicines Evaluation Unit, University Hospital of South Manchester NHS Foundation Trust, Manchester, UK; ^3^The George Institute for Global Health, University of New South Wales, Sydney, Australia;
^4^StatMind, Lund, Sweden;
^5^AstraZeneca, Gothenburg, Sweden

**Methods**

*Study designs and population*

Following the screening and run-in periods, patients were randomised to receive twice-daily (bid) treatment with BUD/FORM 160/4·5 μg (SUN, SHINE, US3, RISE), BUD/FORM 80/4·5 μg (SUN, SHINE, US3), BUD 160 μg plus FORM 4·5 μg (SHINE), BUD 160 μg (SHINE), FORM 4·5 μg (SUN, SHINE, US3, RISE), or placebo (SUN, SHINE).

In SUN, SHINE and US3 confirmed airflow obstruction was defined as a pre-bronchodilator FEV_1_/forced vital capacity (FVC) ratio of <0·7, in addition to a pre-bronchodilator FEV_1_ ≤50% of predicted normal. In RISE, post-bronchodilator FEV_1_/FVC ratio <0·7 and FEV_1_ ≤70% were inclusion criteria. All patients had a history of at least one exacerbation requiring oral corticosteroids (OCS) or antibiotics or both in the 12 months before enrolment. Exclusion criteria included any history of asthma or a history of allergic rhinitis before 40 years of age (SUN, SHINE, US3) or after 18 years of age (RISE).

**Results and Discussion**

The treatment effect on CID was explored in several subgroups based on baseline characteristics, including smoking status, LAMA and ICS use prior to study entry, baseline lung function, exacerbation history, age, gender and total SGRQ score. No consistent treatment effect of BUD/FORM or FORM alone was observed between subgroups across the four different trials (Figure S1).

**Supplementary Tables**

**Table S1. Visit and assessment schedule by study**

| **Visit month** |  | **1** | **2** | **4** | **6** | **9** | **12** |
| --- | --- | --- | --- | --- | --- | --- | --- |
| **SUN**  - **BUD/FORM 160/4∙5 μg (n=494)**  - BUD/FORM 80/4∙5 μg (n=494)  - **FORM 4∙5 μg (n=494)**  - Placebo (n=481) | FEV_1_ | x | x | x | x | x | x |
|  | SGRQ | x | x |  | x |  | x |
| **SHINE**  - **BUD/FORM 160/4∙5 μg (n=277)**  - BUD/FORM 80/4∙5 μg (n=281)  - BUD 160 μg + FORM 4∙5 μg (n=287)  - BUD 160 μg (n=275)  - **FORM 4∙5 μg (n=284)**  - Placebo (n=300) | FEV_1_ | x | x | x | x |  | |
|  | SGRQ | x | x |  | x |  |  |
| **US3**  - **BUD/FORM 160/4∙5 μg (n=404)**  - BUD/FORM 80/4∙5 μg (n=403)  - **FORM 4∙5 μg (n=403)** | FEV_1_ | x | x | x | x | x | x |
|  | SGRQ |  | x | x | x |  | x |
| **RISE**  - **BUD/FORM 160/4∙5 μg (n=606)**  - **FORM 4∙5 μg (n=613)** | FEV_1_ | x | x | x | x |  | |
|  | SGRQ | x | x | x | x |  |  |

The treatment arms used for analyses are marked in bold.
BUD, budesonide; FEV_1_, forced expiratory volume in 1 second; FORM, formoterol; SGRQ, St George’s Respiratory Questionnaire.

**Table S2.** **Demographic and baseline characteristics by study (budesonide/formoterol 160/4·5 μg and formoterol 4·5 μg arms only)**

| **Characteristic** | **SUN (n=989)** | **SHINE (n=561)** | **US3 (n=807)** | **RISE (n=1219)** |
| --- | --- | --- | --- | --- |
| **Age, years** | 63∙0 (40–88) | 63∙3 (41–89) | 63∙1 (40–87) | 63∙5 (40–87) |
| **Male, n (%)** | 631 (63∙8) | 374 (66∙7) | 490 (60∙7) | 698 (57∙3) |
| **Female, n (%)** | 358 (36∙2) | 187 (33∙3) | 317 (39∙3) | 521 (42∙7) |
| **Race, n (%)**  **White**  **Black**  **Asian**  **Other** | 914 (92∙4)  23 (2∙3)  5 (0∙5)  47 (4∙8) | 523 (93∙2)  20 (3∙6)  2 (0∙4)  16 (2∙9) | 667 (82∙8)  33 (4∙1)  10 (1∙2)  96 (11∙9) | 1119 (91∙8)  39 (3∙2)  17 (1∙4)  44 (3∙6) |
| **Former smoker, n (%)** | 573 (57∙9) | 319 (56∙9) | 516 (63∙9) | 655 (53∙7) |
| **Current smoker, n (%)** | 416 (42∙1) | 242 (43∙1) | 291 (36∙1) | 564 (46∙3) |
| **Mean pack-year (range)** | 46∙5 (10–180) | 43∙6 (10–184) | 52∙4 (10–258) | 42∙8 (10–365) |
| **LAMA user, n (%)*** | 217 (21∙9) | 84 (15∙0) | 145 (18∙0) | 396 (32∙5) |
| **LAMA non-user, n (%)*** | 772 (78∙1) | 477 (85∙0) | 662 (82∙0) | 823 (67∙5) |
| **ICS user, n (%)*** | 613 (62∙0) | 244 (43∙5) | 505 (62∙6) | 766 (62∙8) |
| **ICS non-user, n (%)*** | 376 (38∙0) | 317 (56∙5) | 302 (37∙4) | 453 (37∙2) |
| **No. exacerbations in previous year** | 1∙8 (1–13) | 1∙6 (0–8) | 1∙7 (1–12) | 1∙4 (1–7) |
| **Post-FEV_1_, L** | 1∙18 (0∙35–3∙26) | 1∙20 (0∙30–3∙29) | 1∙11 (0∙34–2∙96) | 1∙38 (0∙34–3∙6) |
| **Post-FEV_1_, % predicted** | 38∙9 (13–92) | 39∙3 (10–103) | 37∙7 (12–77) | 48∙7 (16–78) |
| **FEV_1_/FVC ratio** | 0∙49 (0∙20–0∙85) | 0∙48 (0∙20–0∙82) | 0∙47 (0∙16–1∙00) | 0∙49 (0∙19–0∙75) |
| **Neutrophils, x10^9^/L, geometric mean (range)** | 4∙71 (1∙6–21∙6) | 4∙61 (1∙8–19∙4) | 4∙62 (1∙5–26∙4) | N/A† |
| **Eosinophils, x10^9^/L, geometric mean (range)** | 0∙18 (0∙01–1∙47) | 0∙18 (0∙01–1∙01) | 0∙13 (0∙01–2∙51) | N/A† |
| **SGRQ total score** | 54∙9 (7–100) | 54∙9 (14–100) | 57∙5 (6–99) | 46∙7 (0–97) |

Data presented as mean (range) unless otherwise stated.

*Treatment prior to study randomisation/treatment allocation.

†Laboratory data was not assessed in RISE; therefore, no baseline data are available for eosinophils and neutrophils.

FEV_1_, forced expiratory volume in 1 second; FVC, forced vital capacity; ICS, inhaled corticosteroid; LAMA, long-acting muscarinic antagonist; NA, not applicable; SGRQ, St George’s Respiratory Questionnaire.

**Table S3. Demographic and baseline characteristics by occurrence of CID event by study (budesonide/formoterol 160/4·5 μg and formoterol 4·5 μg arms only)**

| **Characteristic** | **SUN** | | **SHINE** | | **US3** | | **RISE** | |
| --- | --- | --- | --- | --- | --- | --- | --- | --- |
|  | **CID event (n=703)** | **No CID**  **(n=286)** | **CID event (n=369)** | **No CID**  **(n=192)** | **CID event (n=585)** | **No CID**  **(n=222)** | **CID event (n=985)** | **No CID**  **(n=234)** |
| **Age, years** | 63∙3 (40–88) | 62∙4 (43–83) | 64∙1 (41–86) | 61∙9 (42–89) | 63∙1 (40–87) | 63∙2 (41–86) | 63∙5 (40–87) | 63∙7 (43–86) |
| **Male, n (%)** | 464 (66∙0) | 167 (58∙4) | 261 (70∙7) | 113 (58∙9) | 358 (61∙2) | 132 (59∙5) | 560 (56∙9) | 138 (59∙0) |
| **Female, n (%)** | 239 (34∙0) | 119 (41∙6) | 108 (29∙3) | 79 (41∙1) | 227 (38∙8) | 90 (40∙5) | 425 (43∙1) | 96 (41∙0) |
| **Race, n (%)**  **White**  **Black**  **Asian**  **Other** | 660 (93∙9)  16 (2∙3)  3 (0∙4)  24 (3∙4) | 254 (88∙8)  7 (2∙4)  2 (0∙7)  23 (8∙0) | 346 (93∙8)  13 (3∙5)  2 (0∙5)  8 (2∙2) | 177 (92∙2)  7 (3∙6)  0 (0∙0)  8 (4∙2) | 477 (81∙7)  22 (3∙8)  7 (1∙2)  78 (13∙4) | 190 (85∙6)  11 (5∙0)  3 (1∙4)  18 (8∙1) | 903 (91∙9)  30 (3∙1)  15 (1∙5)  35 (3∙6) | 216 (92∙3)  9 (3∙8)  2 (0∙9)  7 (3∙0) |
| **Former smoker, n (%)** | 403 (57∙3) | 170 (59∙4) | 217 (58∙8) | 102 (53∙1) | 373 (63∙8) | 143 (64∙4) | 538 (54∙6) | 117 (49∙6) |
| **Current smoker, n (%)** | 300 (42∙7) | 116 (40∙6) | 152 (41∙2) | 90 (46∙9) | 212 (36∙2) | 79 (35∙6) | 447 (45∙4) | 117 (49∙6) |
| **Mean pack-year (range)** | 46∙7 (10–180) | 45∙8 (10–150) | 43∙5 (10–184) | 43∙8 (11–150) | 53∙2 (10–258) | 50∙3 (10–200) | 42∙7 (10–168) | 43∙4 (10–365) |
| **LAMA user, n (%)*** | 171 (24∙3) | 46 (16∙1) | 65 (17∙6) | 19 (9∙9) | 98 (16∙8) | 47 (21∙2) | 313 (31∙8) | 83 (35∙5) |
| **LAMA non-user, n (%)*** | 532 (75∙7) | 240 (83∙9) | 304 (82∙4) | 173 (90∙1) | 487 (83∙2) | 175 (78∙8) | 672 (68∙2) | 151 (64∙5) |
| **ICS user, n (%)*** | 454 (64∙6) | 159 (55∙6) | 160 (43∙4) | 84 (43∙8) | 378 (64∙6) | 127 (57∙2) | 609 (61∙8) | 157 (67∙1) |
| **ICS non-user, n (%)*** | 249 (35∙4) | 127 (44∙4) | 209 (56∙6) | 108 (56∙3) | 207 (35∙4) | 95 (42∙8) | 376 (38∙2) | 77 (32∙9) |
| **No. exacerbations in previous year** | 1∙8 (1–13) | 1∙7 (1–10) | 1∙7 (1–08) | 1∙5 (0–6) | 1∙8 (1–10) | 1∙6 (1–12) | 1∙4 (1–7) | 1∙3 (1–5) |
| **Post-FEV_1_, L** | 1∙18 (0∙35–3∙26) | 1∙18 (0∙35–2∙45) | 1∙18 (0∙30–3∙29) | 1∙26 (0∙38–2∙79) | 1∙08 (0∙34–2∙56) | 1∙20 (0∙37–2∙96) | 1∙38 (0∙34–3∙06) | 1∙37 (0∙46–2∙98) |
| **Post-FEV_1_, % predicted** | 38∙6 (13–92) | 39∙7 (14–77) | 38∙3 (10–103) | 41∙2 (10–73) | 36∙7 (12–74) | 40∙3 (14–77) | 48∙8 (16–74) | 48∙5 (19–78) |
| **FEV_1_ reversibility, %** | 16∙0 (-26–100) | 16∙4 (-36–89) | 17∙4 (-16–147) | 18∙6 (-62–159) | 15∙4 (-22–151) | 19∙8 (-15–73) | 11∙5 (-51–76) | 10∙3 (-50–116) |
| **FEV_1_/FVC ratio** | 0∙48 (0∙20–0∙85) | 0∙51 (0∙21–0∙82) | 0∙48 (0∙24–0∙82) | 0∙50 (0∙20–0∙80) | 0∙46 (0∙16–1∙00) | 0∙51 (0∙25–0∙80) | 0∙49 (0∙22–0∙75) | 0∙49 (0∙19–0∙70) |
| **Neutrophils, x10^9^/L, geometric mean (range)** | 4∙75 (1∙6–21∙6) | 4∙62 (1∙7–12∙2) | 4∙66 (1∙8–19∙4) | 4∙51 (1∙8–12∙8) | 4∙71 (1∙5–26∙4) | 4∙40 (1∙5–14∙7) | N/A† | N/A† |
| **Eosinophils, x10^9^/L, geometric mean (range)** | 0∙177 (0∙01–1∙47) | 0∙190 (0∙01–1∙13) | 0∙179 (0∙02–1∙01) | 0∙168 (0∙01–0∙91) | 0∙136 (0∙01–2∙51) | 0∙126 (0∙01–2∙00) | N/A† | N/A† |
| **SGRQ total score** | 53∙7 (7–95) | 57∙9 (12–100) | 54∙6 (18–100) | 55∙5 (14–97) | 57∙3 (6–99) | 58∙1 (11–94) | 46∙4 (0–96) | 48∙0 (3–97) |

Data presented as mean (range) unless otherwise stated.
*Treatment prior to study randomisation/treatment allocation.
†Laboratory data was not assessed in RISE; therefore, no baseline data are available for eosinophils and neutrophils.
CID, Clinically Important Deterioration; FEV_1_, forced expiratory volume in 1 second; FVC, forced vital capacity; ICS, inhaled corticosteroid; LAMA, long-acting muscarinic antagonist; NA, not applicable; SGRQ, St George’s Respiratory Questionnaire.

**Table S4. Outcome of tests on proportional hazards between treatments**

|  | **P-value for interaction** | | | |
| --- | --- | --- | --- | --- |
| **Study** | **CID** | **Exacerbation** | **FEV_1_** | **SGRQ** |
| **Full study length**  **SUN**  **SHINE**  **US3**  **RISE** | 0∙0064  0∙0962  0∙0007  0∙5703 | 0∙0077  0∙2241  <0∙0001  0∙0819 | 0∙0069  0∙4314  0∙5457  0∙2646 | 0∙0182  0∙0256  0∙0181  0∙6511 |
| **6 months**  **SUN**  **SHINE**  **US3**  **RISE** | 0∙1525  0∙3698  0∙0756  0∙3477 | 0∙0233  0∙2003  0∙0286  0∙0754 | 0∙3283  0∙2601  0∙4130  0∙4283 | 0∙0608  0∙1076  0∙0330  0∙2368 |
| **3 months**  **SUN**  **SHINE**  **US3**  **RISE** | 0∙0583  0∙5435  0∙2676  0∙1853 | 0∙2277  0∙3472  0∙2620  0∙0793 | 0∙4465  0∙4184  0∙9904  0∙5691 | 0∙3328  0∙4070  0∙1459  0∙8388 |

CID, Clinically Important Deterioration; FEV_1_, forced expiratory volume in 1 second; SGRQ, St George’s Respiratory Questionnaire.

**Table S5. Analysis of time to first CID by selected eosinophil cut-offs (pooled analysis; 3-month data)**

| **Cut-off, x10^9^/L (% ≤)** | **Lower stratum** | | | **Upper stratum** | | |
| --- | --- | --- | --- | --- | --- | --- |
|  | **BUD/FORM  160/4·5 μg bid** | **FORM**  **4·5 μg bid** | **HR (95% CI)** | **BUD/FORM  160/4·5 μg bid** | **FORM**  **4·5 μg bid** | **HR (95% CI)** |
| **0∙10 (25∙2)** | 132/285 (46∙3) | 147/291 (50∙5) | 0∙88 (0∙70–1∙12) | 338/857 (39∙4) | 470/853 (55∙1) | 0·59 (0·51–0·68) |
| **0∙15 (44∙8)** | 216/503 (42∙9) | 284/520 (54∙6) | 0∙71 (0∙59–0∙85) | 254/639 (39∙7) | 333/624 (53∙4) | 0·61 (0·52–0·72) |
| **0∙20 (60∙9)** | 284/675 (42∙1) | 391/718 (54∙5) | 0∙67 (0∙58–0∙78) | 186/467 (39∙8) | 226/426 (53∙1) | 0·63 (0·52–0·77) |
| **0∙25 (73∙1)** | 340/818 (41∙6) | 461/852 (54∙1) | 0∙67 (0∙58–0∙77) | 130/324 (40∙1) | 156/292 (53∙4) | 0·61 (0·48–0·77) |
| **0∙30 (79∙9)** | 380/896 (42∙4) | 498/931 (53∙5) | 0∙70 (0∙61–0∙79) | 90/246 (36∙6) | 119/213 (55∙9) | 0·51 (0·39–0·68) |
| **0∙35 (85∙2)** | 401/965 (41∙6) | 524/983 (53∙3) | 0∙68 (0∙59–0∙77) | 69/177 (39∙0) | 93/161 (57∙8) | 0·54 (0·39–0·73) |

Numbers shown: event/total (%).
bid, twice daily; BUD, budesonide; CID, Clinically Important Deterioration; CI, confidence interval; FORM, formoterol; HR, hazard ratio.

**Table S6. Analysis of time to first CID by selected eosinophil cut-offs (pooled analysis; 6-month data)**

| **Cut-off, x10^9^/L (% ≤)** | **Lower stratum** | | | **Upper stratum** | | |
| --- | --- | --- | --- | --- | --- | --- |
|  | **BUD/FORM 160/4·5 μg bid** | **FORM**  **4·5 μg bid** | **HR (95% CI)** | **BUD/FORM 160/4·5 μg bid** | **FORM**  **4·5 μg bid** | **HR (95% CI)** |
| **0∙10 (25∙2)** | 163/285 (57∙2) | 183/291 (62∙9) | 0∙85 (0∙69–1∙06) | 454/857 (53∙0) | 564/853 (66∙1) | 0∙62 (0∙55–0∙70) |
| **0∙15 (44∙8)** | 280/503 (55∙7) | 339/520 (65∙2) | 0∙74 (0∙63–0∙86) | 337/639 (52∙7) | 408/624 (65∙4) | 0∙63 (0∙54–0∙72) |
| **0∙20 (60∙9)** | 376/675 (55∙7) | 470/718 (65∙5) | 0∙71 (0∙62–0∙81) | 241/467 (51∙6) | 277/426 (65∙0) | 0∙64 (0∙54–0∙76) |
| **0∙25 (73∙1)** | 451/818 (55∙1) | 554/852 (65∙0) | 0∙71 (0∙62–0∙80) | 166/324 (51∙2) | 193/292 (66∙1) | 0∙60 (0∙48–0∙73) |
| **0∙30 (79∙9)** | 499/896 (55∙7) | 600/931 (64∙4) | 0∙73 (0∙64–0∙82) | 118/246 (48∙0) | 147/213 (69∙0) | 0∙51 (0∙40–0∙65) |
| **0∙35 (85∙2)** | 527/965 (54∙6) | 636/983 (64∙7) | 0∙70 (0∙62–0∙79) | 90/177 (50∙8) | 111/161 (68∙9) | 0∙55 (0∙41–0∙73) |

Numbers shown: event/total (%).
bid, twice daily; BUD, budesonide; CID, Clinically Important Deterioration; CI, confidence interval; FORM, formoterol; HR, hazard ratio.

**Supplementary Figures**

**Figure S1. Summary of CID events by subgroup and by study: a) patient demographics; b) disease history and c) lung function**

**a)**

**
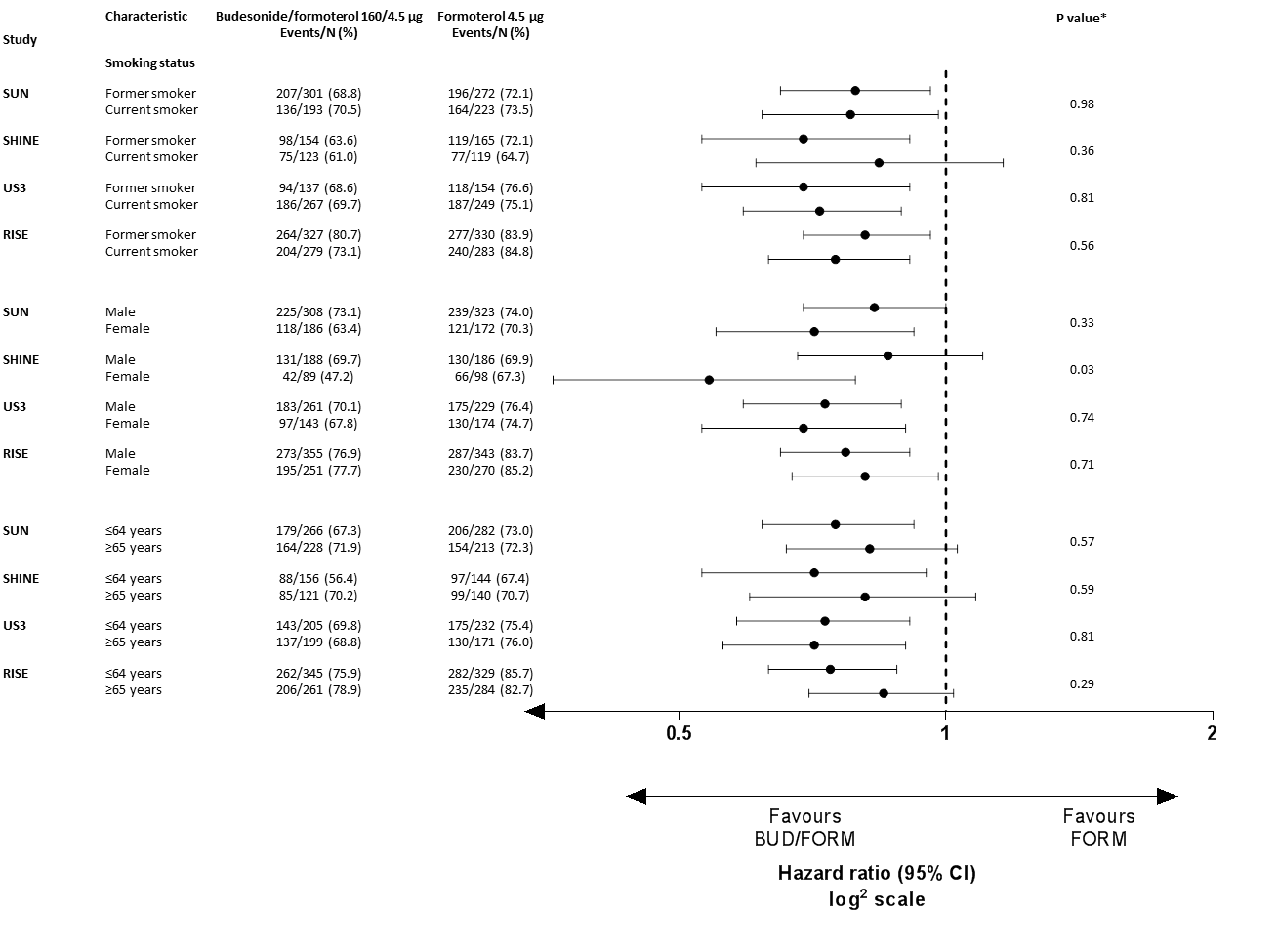
**

*P-value is the test of interaction between treatment and each subgroup unadjusted for multiplicity.
CID, Clinically Important Deterioration; CI, confidence interval; HR, hazard ratio.

**b)**

**
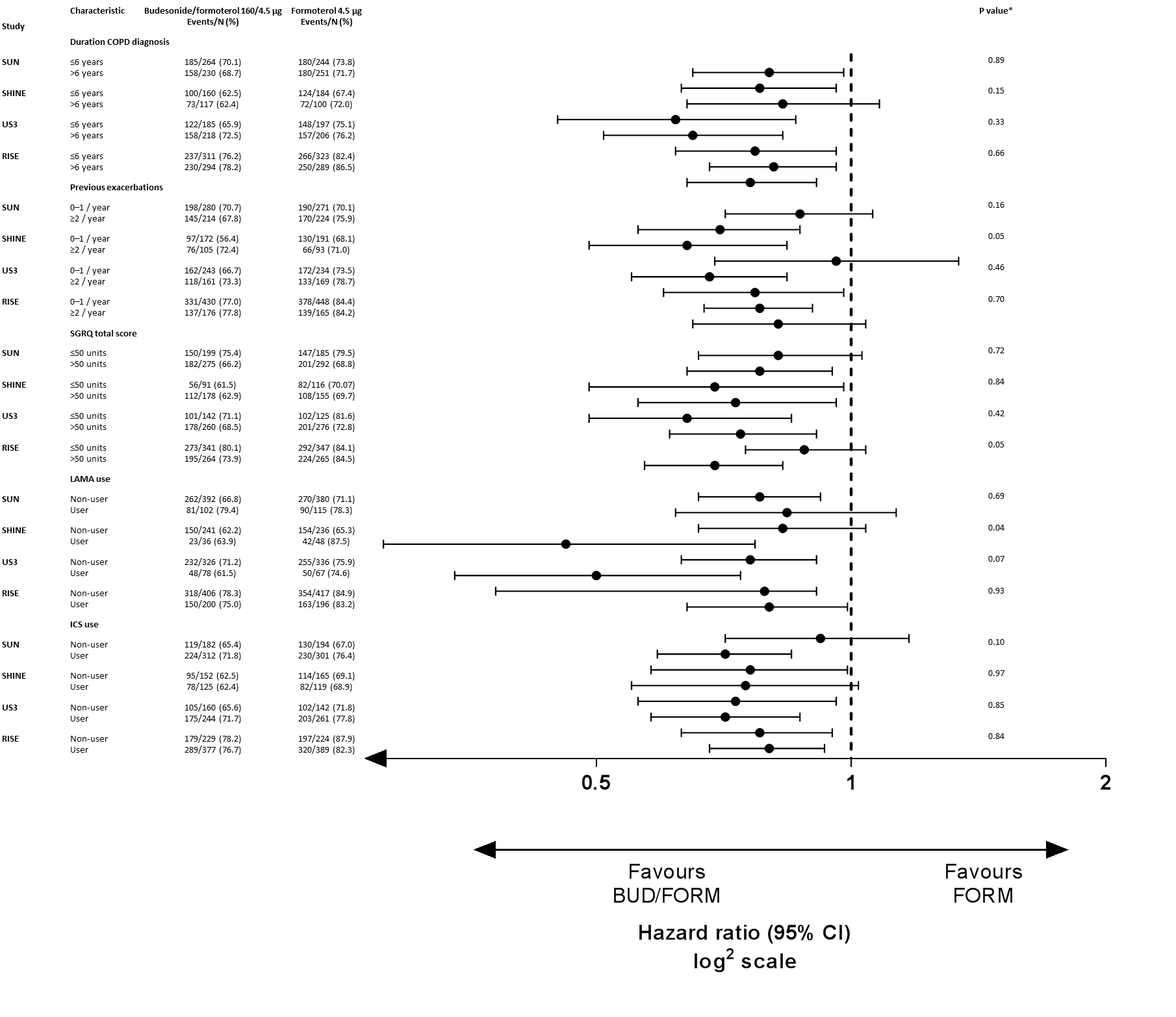
**

*P-value is the test of interaction between treatment and each subgroup unadjusted for multiplicity.
CID, Clinically Important Deterioration; CI, confidence interval; COPD, chronic obstructive pulmonary disease; HR, hazard ratio; ICS, inhaled corticosteroid; LAMA, long-acting muscarinic antagonist; SGRQ, St George’s Respiratory Questionnaire.

**c)**

**
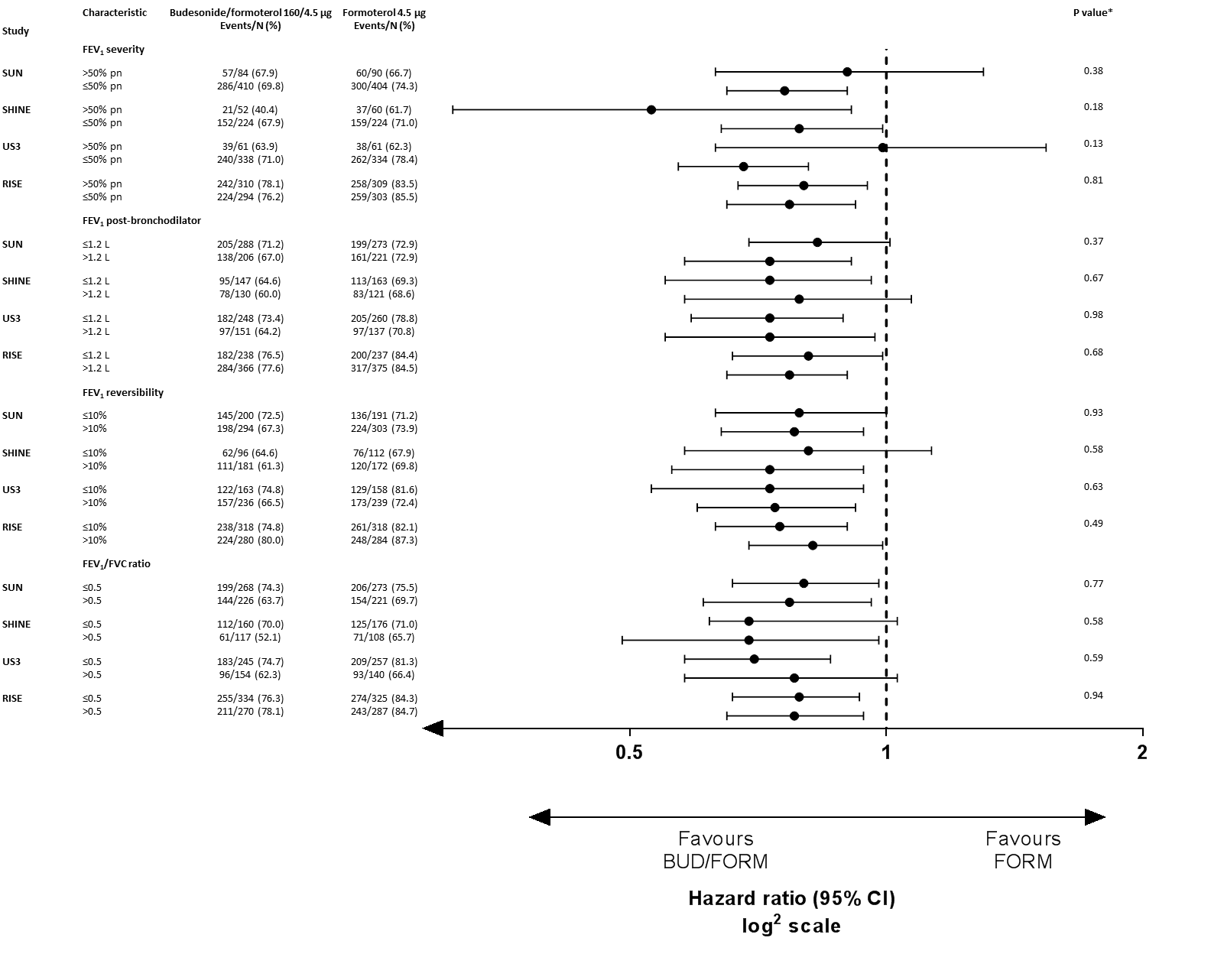
**

*P-value is the test of interaction between treatment and each subgroup unadjusted for multiplicity.
CID, Clinically Important Deterioration; CI, confidence interval; COPD, chronic obstructive pulmonary disease; FEV_1_, forced expiratory volume in 1 second; FVC, forced vital capacity; HR, hazard ratio.

**Figure S2. Forest plot indicating treatment effect of budesonide (BUD) added to formoterol (FORM) on individual components of CID by study, a) full study duration, b) 6 months and c) 3 months**

**a)**

**
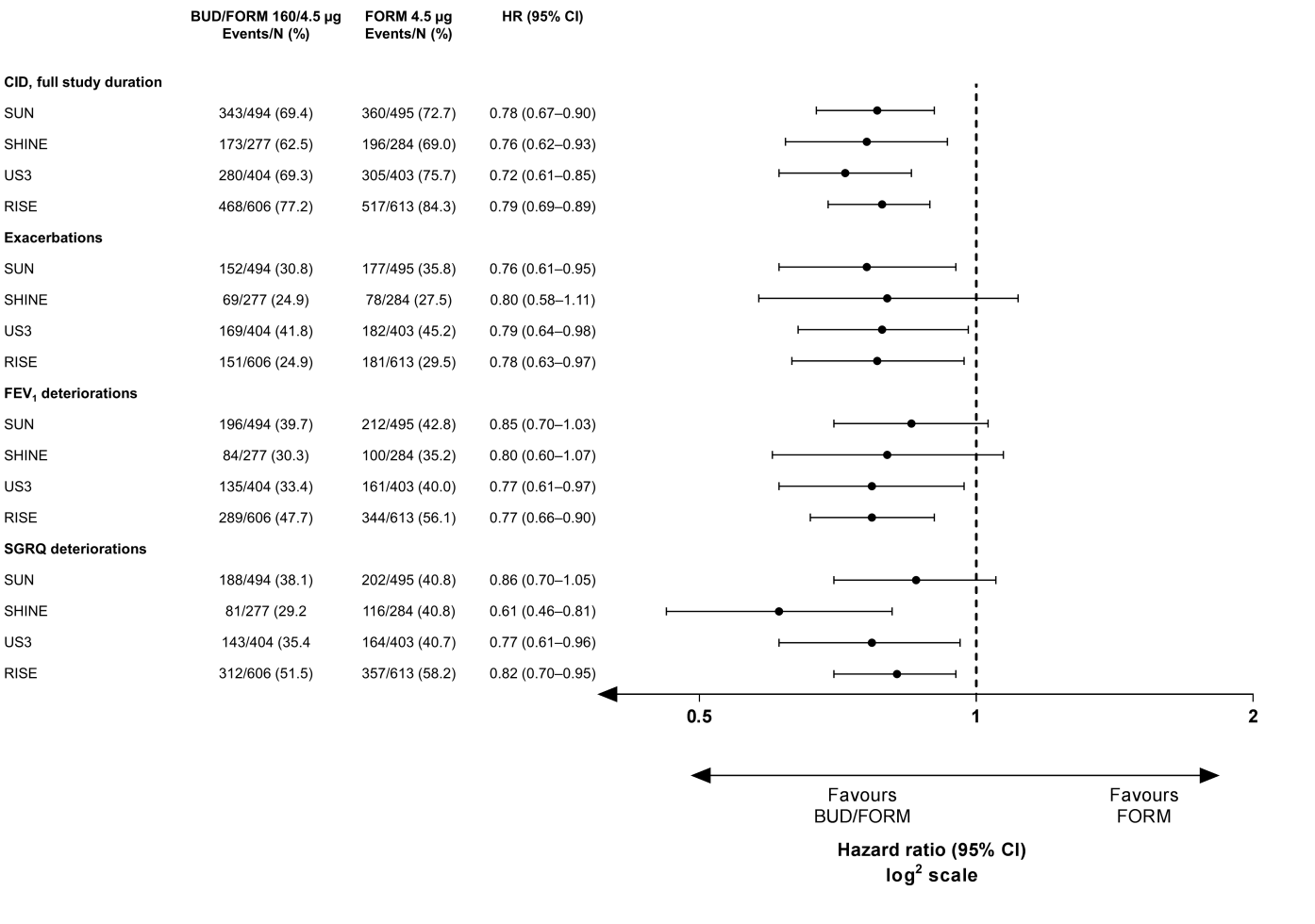
**

CID, Clinically Important Deterioration; CI, confidence interval; FEV_1_, forced expiratory volume in 1 second;
HR, hazard ratio; SGRQ, St George’s Respiratory Questionnaire.

**b)**

**
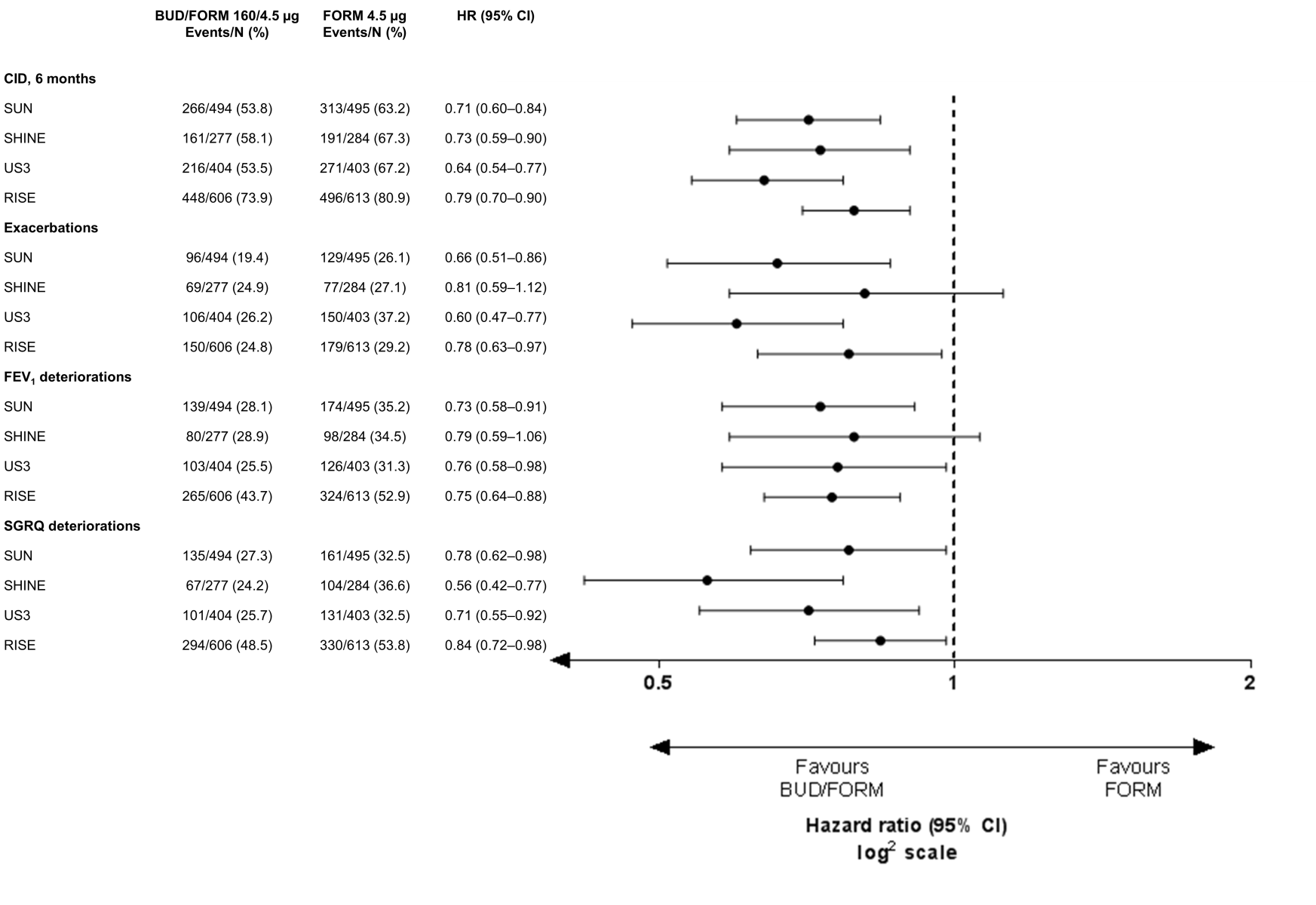
**

CID, Clinically Important Deterioration; CI, confidence interval; FEV_1_, forced expiratory volume in 1 second;
HR, hazard ratio; SGRQ, St George’s Respiratory Questionnaire.

**c)**

**
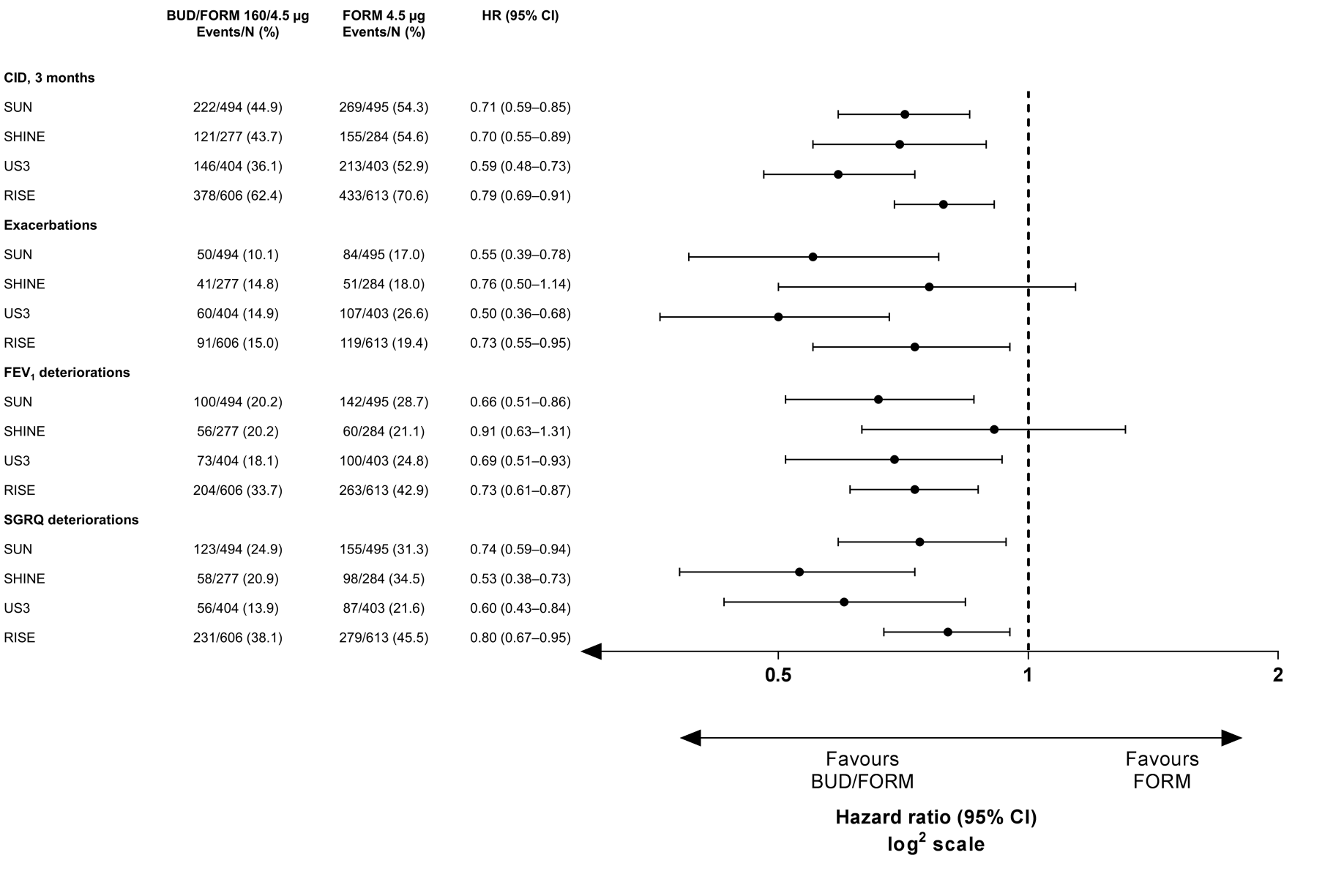
**

CID, Clinically Important Deterioration; CI, confidence interval; FEV_1_, forced expiratory volume in 1 second;
HR, hazard ratio; SGRQ, St George’s Respiratory Questionnaire.

**Figure S3. Hazard ratios (HRs) by eosinophil cut-off in pooled analysis of SUN, SHINE and US3 for a) CID, b) exacerbations, c) FEV_1_ and d) SGRQ**


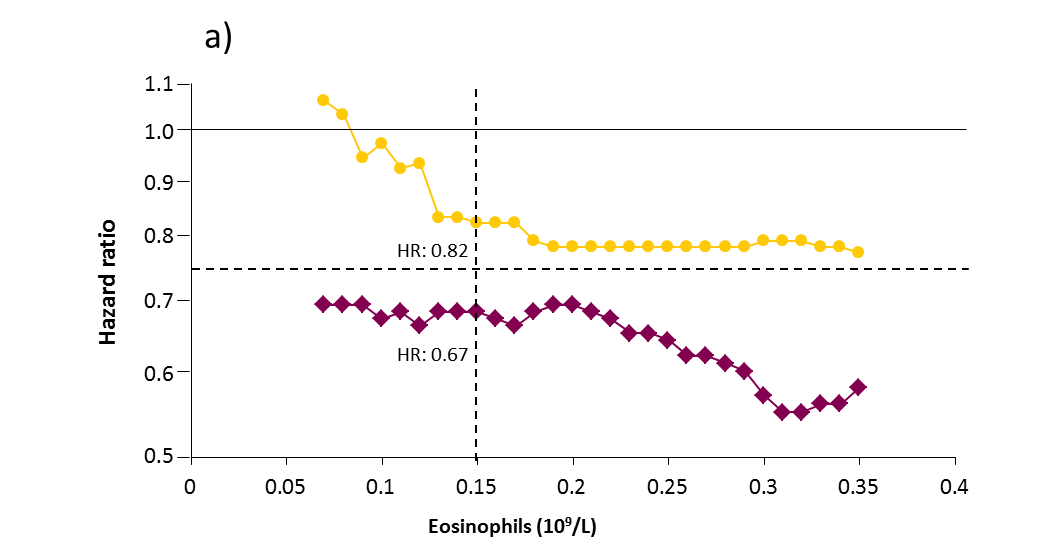


**
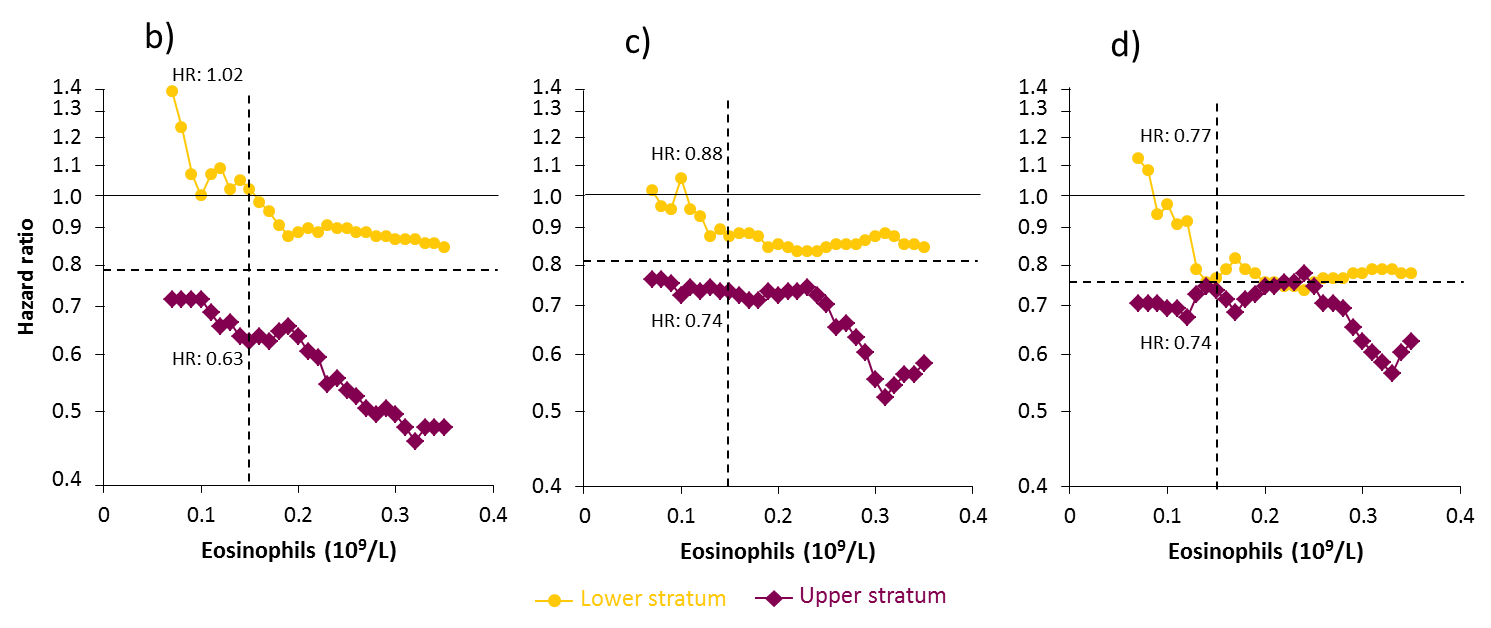
**

Note: The solid line represents HR of 1·0, while the dotted line represents treatment effect for the whole population.
At each individual eosinophil count plotted, HR is calculated for the lower stratum in yellow (indicating the mean HR for all patients with an eosinophil count at or below cut-off) and the upper stratum in purple (indicating the mean HR for all patients above the cut-off). Example HR presented for cut-off at 0·15 x10^9^/L (vertical dotted line).
CID, Clinically Important Deterioration; FEV_1_, forced expiratory volume in 1 second; SGRQ, St George’s Respiratory Questionnaire.

**Figure S4. Effect size by eosinophil cut-off in pooled analysis of SUN, SHINE and US3 for a) 6 months and b) 3 months**


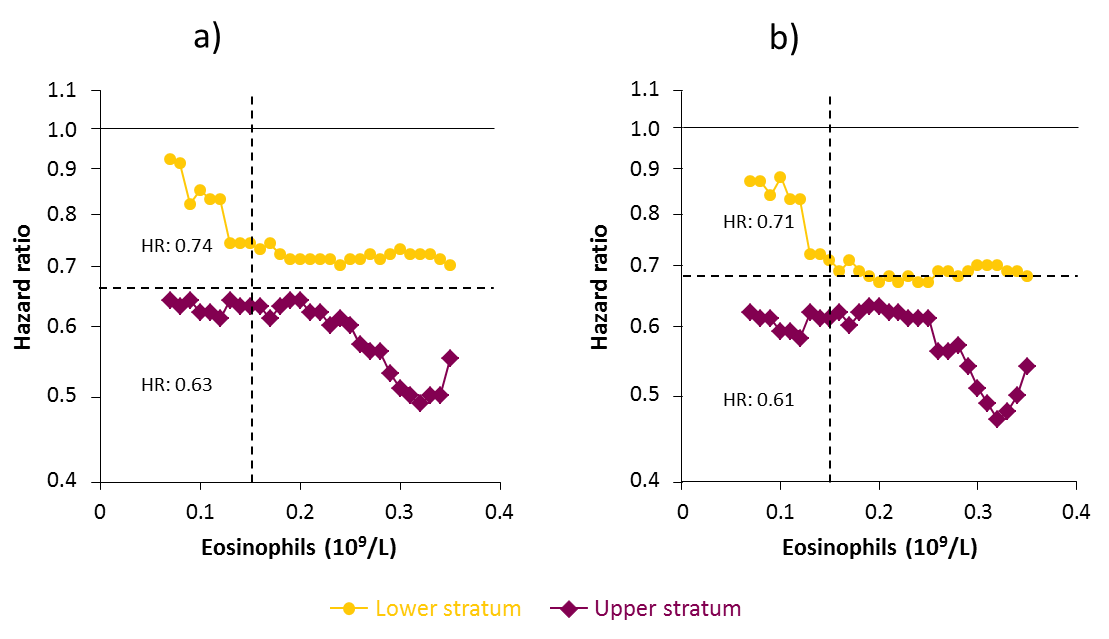


Note: The solid line represents hazard ratio (HR) of 1·0, while the dotted line represents treatment effect for the whole population.
At each individual eosinophil count plotted, HR is calculated for the lower stratum in yellow (indicating the mean HR for all patients with an eosinophil count at or below cut-off) and the upper stratum in purple (indicating the mean HR for all patients above the cut-off). Example HR presented for cut-off at 0·15 x10^9^/L (vertical dotted line).

**Figure S5. Proportion of patients with CID events, by study**

**
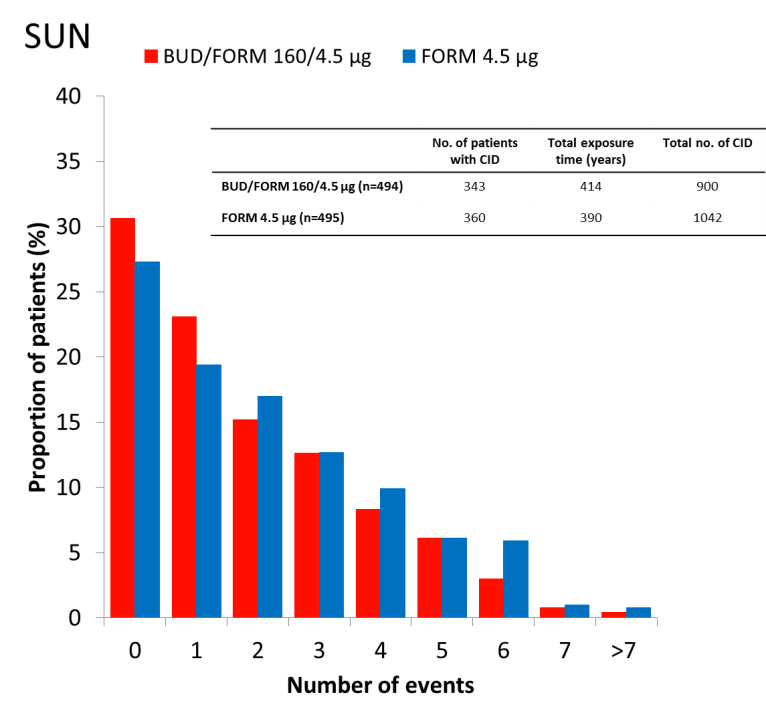
**

**
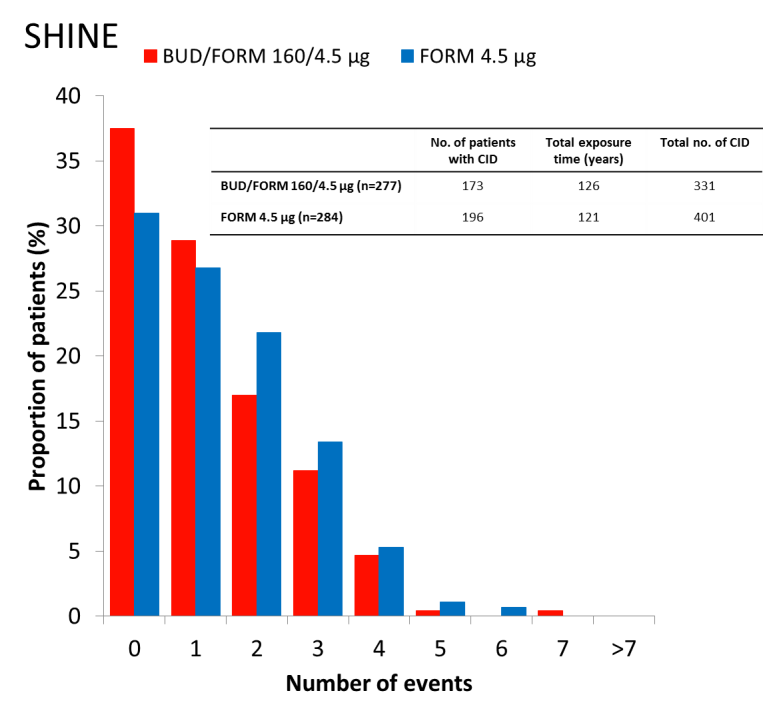
**

**
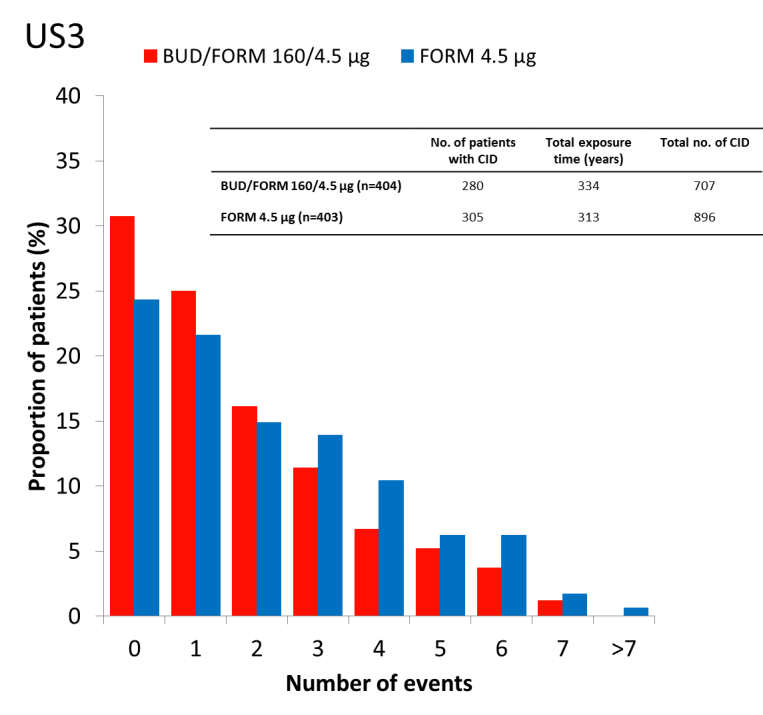
**

**
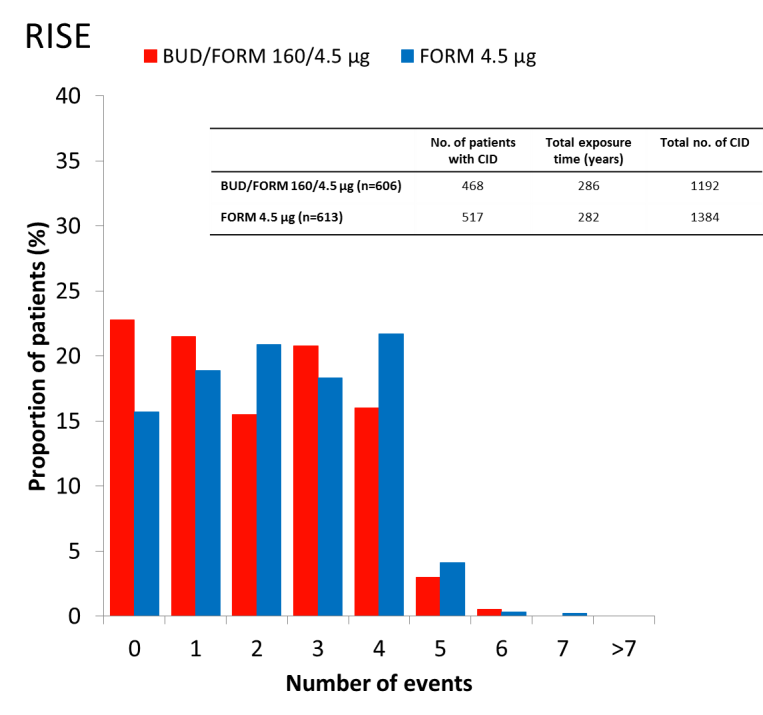
**

BUD, budesonide; CID, Clinically Important Deterioration; FORM, formoterol.

**Figure S6. Forest plot for rate ratio for CID and individual components, by study**

**
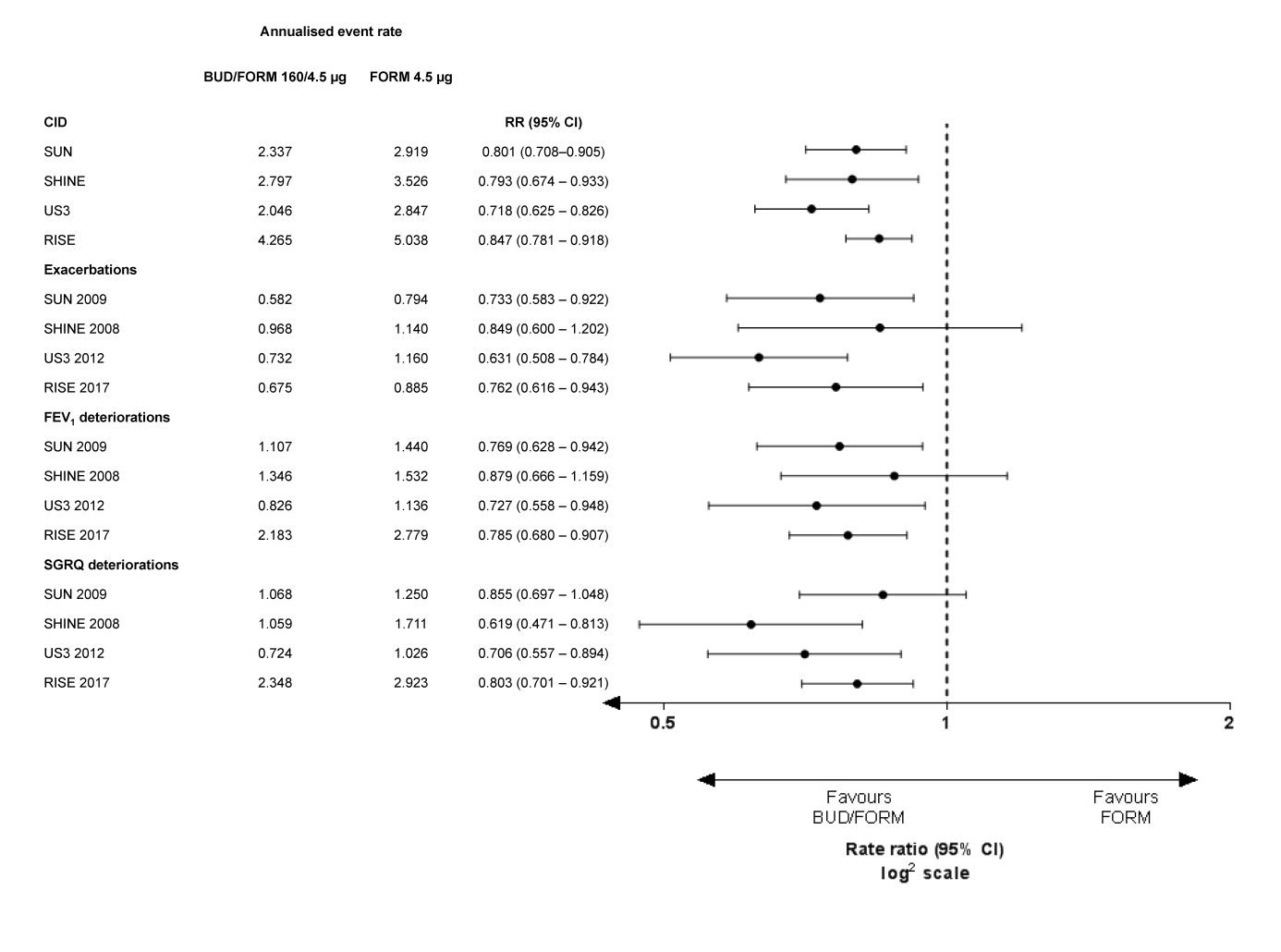
**

Negative binomial models on number of CID events adjusting for treatment and country and time at risk
as offset.

CID, Clinically Important Deterioration; FEV_1_, forced expiratory volume in 1 second; RR, rate ratio; SGRQ, St George’s Respiratory Questionnaire.

**Figure S7. Risk ratios by eosinophil cut-off in pooled analysis of SUN, SHINE and US3**

**
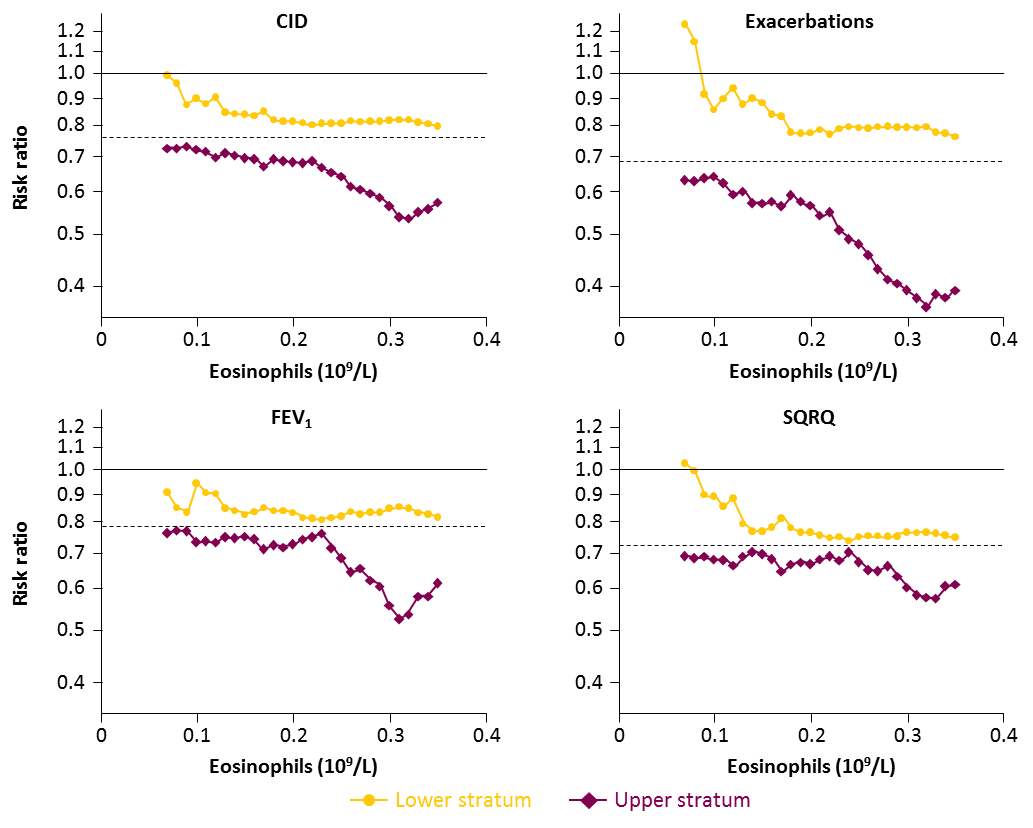
**

Note: The solid line represents risk ratio (RR) of 1·0, while the dotted line represents risk ratio for the whole population.
At each individual eosinophil count plotted, RR is calculated for the lower stratum in yellow (indicating the mean RR for all patients with an eosinophil count at or below cut-off) and the upper stratum in purple (indicating the mean RR for all patients above the cut-off).
